# A Randomised Placebo-Controlled Trial of a Traditional Chinese Herbal Formula in the Treatment of Primary Dysmenorrhoea

**DOI:** 10.1371/journal.pone.0000719

**Published:** 2007-08-15

**Authors:** Lan Lan Liang Yeh, Jah-Yao Liu, Kao-Si Lin, Yu-Shen Liu, Jeng-Min Chiou, Kung-Yee Liang, Te-Feng Tsai, Li-Hsiang Wang, Chiung-Tong Chen, Ching-Yi Huang

**Affiliations:** 1 Division of Clinical Research, National Health Research Institutes, Zhunan, Taiwan; 2 Department of Obstetrics and Gynaecology, Tri-Service General Hospital of National Defense Medical Centre, Taipei, Taiwan; 3 Department of Chinese Medicine, Tri-Service General Hospital of National Defense Medical Centre, Taipei, Taiwan; 4 Section of Obstetrics and Gynaecology, Taipei Municipal Hospital Zhongxiao Branch, Taipei, Taiwan; 5 Institute of Statistical Science of Academia Sinica, Taipei, Taiwan; 6 Division of Biostatistics and Bioinformatics, National Health Research Institutes, Zhunan, Taiwan; 7 Section of Chinese Medicine, Taipei Municipal Hospital Zhongxiao Branch, Taipei, Taiwan; 8 Division of Biotechnology and Pharmaceutical Research, National Health Research Institutes, Zhunan, Taiwan; University of Southampton, United Kingdom

## Abstract

**Background:**

Most traditional Chinese herbal formulas consist of at least four herbs. Four-Agents-Decoction (Si Wu Tang) is a documented eight hundred year old formula containing four herbs and has been widely used to relieve menstrual discomfort in Taiwan. However, no specific effect had been systematically evaluated. We applied Western methodology to assess its effectiveness and safety for primary dysmenorrhoea and to evaluate the compliance and feasibility for a future trial.

**Methodology/Principal Findings:**

A randomised, double-blind, placebo-controlled, pilot clinical trial was conducted in an ad hoc clinic setting at a teaching hospital in Taipei, Taiwan. Seventy-eight primary dysmenorrheic young women were enrolled after 326 women with self-reported menstrual discomfort in the Taipei metropolitan area of Taiwan were screened by a questionnaire and subsequently diagnosed by two gynaecologists concurrently with pelvic ultrasonography. A dosage of 15 odorless capsules daily for five days starting from the onset of bleeding or pain was administered. Participants were followed with two to four cycles for an initial washout interval, one to two baseline cycles, three to four treatment cycles, and three follow-up cycles. Study outcome was pain intensity measured by using unmarked horizontal visual analog pain scale in an online daily diary submitted directly by the participants for 5 days starting from the onset of bleeding or pain of each menstrual cycle. Overall-pain was the average pain intensity among days in pain and peak-pain was the maximal single-day pain intensity. At the end of treatment, both the overall-pain and peak-pain decreased in the Four-Agents-Decoction (Si Wu Tang) group and increased in the placebo group; however, the differences between the two groups were not statistically significant. The trends persisted to follow-up phase. Statistically significant differences in both peak-pain and overall-pain appeared in the first follow-up cycle, at which the reduced peak-pain in the Four-Agents-Decoction (Si Wu Tang) group did not differ significantly by treatment length. However, the reduced peak-pain did differ profoundly among women treated for four menstrual cycles (2.69 (2.06) cm, mean (standard deviation), for the 20 women with Four-Agents-Decoction and 4.68 (3.16) for the 22 women with placebo, *p* = .020.) There was no difference in adverse symptoms between the Four-Agents-Decoction (Si Wu Tang) and placebo groups.

**Conclusion/significance:**

Four-Agents-Decoction (Si Wu Tang) therapy in this pilot post-market clinical trial, while meeting the standards of conventional medicine, showed no statistically significant difference in reducing menstrual pain intensity of primary dysmenorrhoea at the end of treatment. Its use, with our dosage regimen and treatment length, was not associated with adverse reactions. The finding of statistically significant pain-reducing effect in the first follow-up cycle was unexpected and warrants further study. A larger similar trial among primary dysmenorrheic young women with longer treatment phase and multiple batched study products can determine the definitive efficacy of this historically documented formula.

**Trial Registration:**

Controlled-Trials.com ISRCTN23374750

## Introduction

Finding effective traditional Chinese herbal medicines has now become one of the interests of medicine, pharmacology, and agricultural biotechnology worldwide. The thousand-year-old Chinese herbal medicine, mysterious to many, is usually composed of 3–5 herbs in a formula or prescription for treatment of illness, according to several versions of ancient pharmacopoeia. Despite the fact of a long history of human use, a clinical trial for establishing evidence-based medicine has been thought essential. Some clinical trials provide evidence for the effectiveness of traditional Chinese medicine [1], [2], but at present, consistent and systematic evidence-based data are still lacking to support the efficacy or safety and to separate the effects of the active ingredients from those of their presumed complex compositions.

The decision to test Four-Agents-Decoction (Si Wu Tang) resulted from the responses of three surveys of employees in a research institute and women in a college for two consecutive years, each consisting of an open-end questionnaire, administered between 2000 and 2002 (unpublished data) on the prevalence of using traditional Chinese medicine. Among the 227 research institute employees who responded to the survey, Four-Agents-Decoction ranked first as the most frequently used Chinese medicine in the past year, and over half of the dysmenorrheic college students had taken this formula for prophylaxis or relief of menstrual discomfort.

Four-Agents-Decoction (Si Wu Tang) is originally listed in the Prescriptions of People's Welfare Pharmacy (in Chinese) as a remedy for nourishing the blood and has been used as a basic formula in traditional Chinese medicine for treating women's illnesses since the Sung Dynasty (12th century). The formula is composed of dry roots of four plants native to Mainland China: prepared Radix *Rehmanniae praeparata* (Soe Dee Huang), Radix *Paeoniae Alba* (Bai Sau), Radix *Angelicae Sinensis* (Dang Guay), *and* Rhizoma *Ligustici Chuanxiong* (Tsuan Chyong), which can be obtained at Chinese medicine shops without seeking consultation from traditional Chinese medicine practitioners. Traditionally, this formula is used as a concoction.

This formula is worth studying, as non-specified dysmenorrhoea is one of the most common gynaecological complaints in young women. The prevalence rate for dysmenorrhoea varies worldwide; estimates were from 44% in China [3], 51% in Singapore [4], 52–64% in Mexico [5], 60–80% in the United States [6]–[8], 73% in Sweden [9], 80% in Western Australia [10], to 45–95% in Britain [11]. An estimated 42–51% of dysmenorrheic women reported missing either school or work due to severe menstrual pain [8]–[9]. There is a subset of women who do not respond to conventional treatment and turn to alternative medicine or therapy [12]. But information on the use of alternative treatments is not clearly known. In spite of several effective therapies, such as analgesics and oral contraceptives [13]–[15], the morbidity from dysmenorrhoea remains a challenge to public health worldwide.

In traditional Chinese medicine dysmenorrhoea is termed as “painful menstruation,” and is not sub-classified as primary dysmenorrhoea or secondary dysmenorrhoea. Menstrual pain is the major symptom of primary dysmenorrhoea. In this study we aimed for young women with primary dysmenorrhoea, who were differentiated from secondary dysmenorrhoea by noninvasive pelvic ultrasonography and with normal serum level of CA-125 at 35 U/ml as reference [16].

We did not find any published reports or warnings on the use of Four-Agents-Decoction (Si Wu Tang). A post-market clinical trial (phase IV) was thus applied to examine effectiveness by the improvement of primary dysmenorrhoea and assess adverse reactions by the routine clinical examination in conventional medicine and the symptoms in traditional Chinese medicine.

## Methods

The protocol for this trial and supporting CONSORT checklist are available as supporting information; see Protocol S1 and Checklist S1.

### Participants

**Figure 1:**
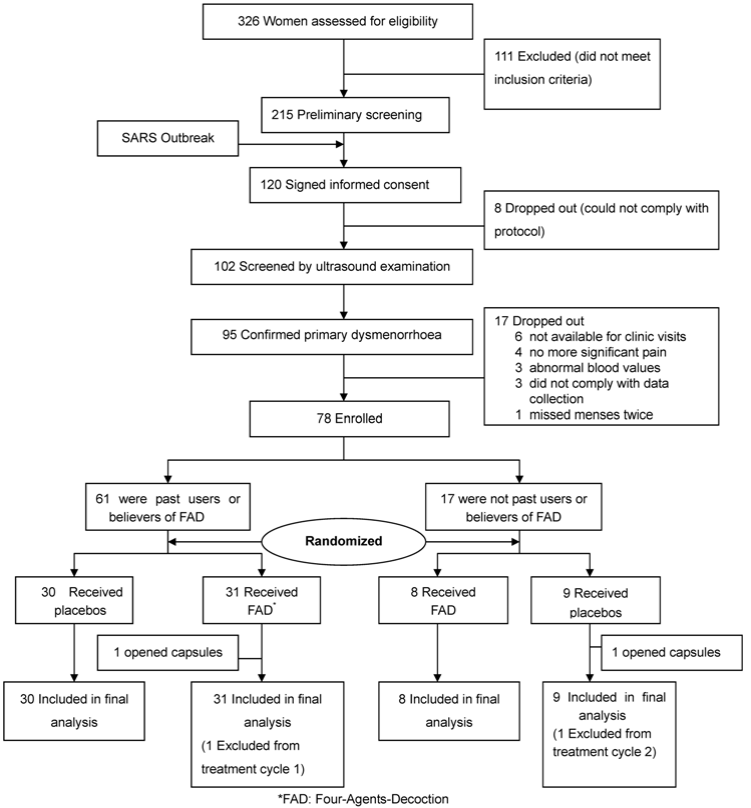
Flow of participants through the enrollment and treatment.

Ethical approval for this study was obtained from the Human Ethics Committee of National Health Research Institutes, Taiwan. We recruited and selected our participants at the Study Centre through the following procedures: (a) *recruitment*: from January to March of 2003, 453 young dysmenorrheic women who informed us their interests in our poster or flyer distributed in colleges over the Taipei Metropolitan area, and of those, 326 returned our questionnaire regarding their history of health, menstruation pattern, pain intensity, and the experience with Four-Agents-Decoction (Si Wu Tang); (b) *eligibility screen*: 215 respondents aged 18 years or older were screened if they had cycles lasting 21–35 days with the actual menses periods lasting three to seven days, and they experienced at least 4 consecutive painful periods in the past six months with the pain starting one day before or on the day of onset of bleeding. Moreover, the women were not taking oral contraceptive pills and agreed to refrain from sexual activity during the study due to any confounding effect on pain and early withdrawal by pregnancy, and they had no severe gastrointestinal, gynaecological or autoimmune diseases, or gynaecological surgery, including pregnancy; (c) *consent to participate*: 120 respondents provided written, informed consent, which included agreeing to take nothing but analgesics and study product provided by the centre for the pain, to have examinations by both two gynaecologists and four traditional Chinese medicine physicians, to have blood drawn, and to have remaining blood specimens stored after the tests, and to observe menstruation; (d) *washout*: established baseline with provided analgesics (ibuprofen 400mg, four times daily for three days) for pain relief for 2–4 menstrual cycles; (e) *confirmation*: 95 primary dysmenorrheic women were confirmed after ruling out an organic lesion with a pelvic ultrasound examination on the abdomen by both study gynaecologists and serum CA-125 level less than 35 U/ml or decreased to 40 U/ml from the second blood draw; and (f) *final eligibility*: 81 women were eligible after excluding those with abnormal hematological and biochemical indices of hepatic or renal function; (g) *enrollment*: 78 young women were enrolled after three women subsequently stopped participation during the baseline period due to getting married, no longer having painful menstruation, or not being willing to take study capsules (Figure 1).

### Interventions

We purchased the study products from a manufacturer with Good Manufacturing Practice. This custom-made Four-Agents-Decoction (Si Wu Tang) was encapsulated with granules from concentrated decoction made by water extraction in 1∶13 ratio from single batched roots of the four plants in equal proportions: prepared Radix *Rehmanniae praeparata* (Soe Dee Huang), Radix *Paeoniae alba* (Bai Sau), Radix *Angelicae sinensis* (Dang Guay), and Rhizoma *Ligustici chuanxiong* (Tsuan Chyong), as prepared according to the original pharmacopoeia. The plant origins in China were known to the buyer of the pharmaceutical company. According to the habitual usage of this formula and the comparisons of the decoction made from raw materials with the commercially available granules of concentrated decoction, the concentrations of two quality control indicators-paeoniflorin and ferulic acid**-**within the ranges requested by the Principal Investigator were met. Provided by the pharmaceutical company, the final product was free of *E. coli* and *Salmonella* and the levels of heavy metals were 0.45 ppm for lead, 0.14 ppm for arsenic, 0.01 ppm for cadmium, and 0.02 ppm for mercury, all within regulated limits (20, 5, 0.5, and 0.5 ppm, respectively). Every capsule was sealed and secured with a band at the interface by a capsule supply company to ensure concealment of the aroma from the materials (Figure S1). The identical looking placebo capsules were filled with a powder mixture of cornstarch and caramel. Each capsule weighed approximately 500 mg.

Regimens of five capsules were packaged in aluminum packets for easier handling and to prevent Four-Agents-Decoction (Si Wu Tang) from moisture exposure. Clinic visit number and case number were marked on each packet in both text and bar code. The bar code with the number of unused capsules from used packets was scanned into the database after each treatment cycle.

We determined the dosage of 15 capsules daily for five days from the onset of bleeding or pain after taking considerations: (a) habitual usages provided from the three surveys, (b) literature reports, (c) a comparison of the concentrations of two indices, paeoniflorin and ferulic acid, in a one-day dose of a commercially available powder with the decoction made from raw materials, (d) information from the study's traditional Chinese medicine physicians, and (e) input from the study's leading gynaecologist. For easier intake, we instructed the women to take the capsules with warm water three times. A treatment cycle entailed a total of 75 capsules. The exact number of capsules taken each day was recorded in the online diary, and any unfinished capsules were brought back to the study nurse at the next clinic visit.

Participants' mean menstrual cycle length at baseline was 27 and ranged from 21 to 43 days. However, some women's cycles had shortened after participating in the trial. We initially set the treatment duration for three menstrual cycles [12]. In order to have the post-treatment examination by the same six study physicians, the treatment duration was imperatively extended to four menstrual cycles for women having shortened cycles and for those out of town for month-long winter break. We received informed consent for additional treatment cycle from these women. All women then were followed for three cycles after completing treatment.

### Objectives

We hypothesized that Four-Agents-Decoction (Si Wu Tang) had a beneficial effect to treat primary dysmenorrhoea. Using a scientific, not market-driven approach applying Good Clinical Practice guidelines established by the Department of Health, Taiwan (Publication No. 85067127), the objective of this pilot study was to gather preliminary data on compliance, feasibility, safety, and effectiveness to power a similar future trial.

### Outcomes

#### (1) Primary outcome: visual analogue pain scale (VAS)

We collected the primary outcome data during the first five days of each menstrual cycle using a horizontal unmarked visual analogue scale (VAS) of 0–10 cm (0 cm representing no pain; 10 cm, severe pain) [17] with a sliding bar on the scale posted on our Web site for participants to gauge their pain feeling. The online scale matched 1000 scales in our database in order to capture the maximal pain and detect small differences. The participants recorded the pain intensity they experienced right before taking ibuprofen (400 mg), which was provided by the Study Centre for unbearable menstrual pain after holding it off.

#### (2) Other pain measures

In order to compare with the results from other studies assessing the pain by verbal scale, we also collected data on a 4-point pain scale (no pain, mild pain, moderate pain, and severe pain) from the online questionnaire at each cycle. Dichotomous responses (no pain or pain) were then grouped from this scale.

#### (3) Confounders

Data regarding the type and frequency of the intake of all drugs and the use of other remedies, as possible confounders, were collected along with the VAS pain intensity in the electronic diary.

#### (4) Adverse reactions or events

At the time of the pre-treatment ultrasound screening and the post-treatment (end of treatment) examination, a total of 20 ml of venous blood was obtained for safety evaluation. Creatinine, BUN (urea nitrogen), and uric acid were assessed for renal function; AST (aspartate aminotransferase) and ALT (alanine aminotransferase), for liver function; iron, TIBC (total iron binding capacity), hemoglobin, and hematocrit, for anemic condition; and mercury, arsenic, lead, cadmium, and copper, for heavy metal toxicity.

Possible adverse reactions noted by traditional Chinese medicine physicians were collected: inner heat reaction, (referring to fire energy or Huo Chi in Pinyin, a symptom used in traditional Chinese medicine, which corresponds to inflammation in Western conventional medicine), such as blisters and pimples, and disturbed menstruation, such as amenorrhea and menorrhagia. Participants evaluated the latter symptom using a balance and pads of three sizes supplied by the Study Centre and reported the weight of soiled pads by size in the electronic diary. In addition, any other discomforts or illnesses and the number of days missing work or school were collected.

### Sample size

We positioned this trial as a pilot study. Very few studies of non-conventional treatment for primary dysmenorrhoea contain data that can be referred for sample size calculation. We found 40 participants in one comparable study [18], and 100 participants in another [19]. Clinical trials on conventional analgesics were thus referred, in which the average pain reduction rate was 70% in the treatment group and 30% in the placebo group. With 90% power and 5% type I error (two-sided), we required 28 participants for each arm of this trial. Therefore, 100 participants were to be enrolled to allow for a 25% dropout rate.

### Randomization—Sequence generation

The 78 dysmenorrheic women were first stratified by their past experience or belief in the effectiveness of Four-Agents-Decoction as reported in the recruitment questionnaire [20]. In each stratum random codes with a permuted block randomization scheme were generated by computer.

### Randomization—Allocation concealment

The participants were not given their group assignment code. The study physicians did not share their own examination results, did not know the identity of the participants, did not handle the study products, and did not know the assigned treatment. Two sets of 78 sealed opaque envelopes containing each individual's treatment assignment were prepared; one set was for the leading gynaecologist to keep for emergency care and the other was kept with Principal Investigator. The two envelopes remained sealed until data analysis.

### Randomization—Implementation

All clinic visits for the 78 participants were arranged at the Study Centre; the participants were randomized into Four-Agents-Decoction or placebo groups based on the order of their chosen date and the arrival time for their post-screening clinic. A total of 39 women were allocated to each treatment and placebo group, according to the computer generated allocation sequence by study statistician.

### Blinding

The appearances of the study product and placebo capsules were identical and no aroma was detected from either. Achievement of blindness was validated before the trial in a group of 39 volunteers (unpublished data). At the completion of treatment, we conducted a simple survey asking the participants to guess whether they were in the study product or placebo group in order to evaluate the extent of study blindness.

### Statistical methods

All data were stratified by treatment group and examined separately. Study outcome was visual analog pain intensity given as mean score (cm) and 95% confidence interval. Peak-pain was the maximal single-day pain intensity and overall-pain was the average pain intensity among days in pain. Data of the cycle adjacent to the first treatment cycle was set as baseline. As menstrual pain on the most intense day is considered unbearable for many dysmenorrheic women, we focused our analysis on the peak-pain.

Our outcome data were repeated measurements and correlated across successive cycle points over the entire trial. The model fit by generalized estimating equations (GEE) [21]–[22] is able to account for longitudinal and correlated response data [23]. We tested the nonlinear relationship and possible interaction effects between treatments and cycles from baseline to the end of treatment with the data based on individual trajectories of peak-pain measurements. The GENMOD procedure in SAS (version 8.2 SAS institute, Inc., Cary, North Carolina, U.S.A.) was used for the modeling. The values for the variable “*cycle*” were set from 0 to 4 cycles according to the order of treatment cycle from baseline, respectively, and the indicator variable “*treatment*” was used to distinguish the two groups, with the placebo group as the reference. The explanatory variables in the models consisted of the indicator variables “*treatment*”, “*cycle*”, and the quadratic term “*cycle^2^*” for nonlinear trends, as well as their interaction terms “*cycle*×*treatment*” and “*cycle^2^*×*treatment*”. The treatment effect was characterized using the two interaction terms.

We compared the peak-pain intensity by t-test for at each treatment and follow-up cycle between the two treatment groups and for those with three or four treatment cycles. Statistical significance was determined at *p*≤.05. Over-all pain intensity distribution throughout the trial was presented and the same comparisons were performed between the two treatment groups.

## Results

### Participant flow

Figure 1 summarizes the enrollment, treatment allocation, and data analysis for the 78 participants with primary dysmenorrhoea. Two women were withdrawn because they opened the capsules for easier intake: one was from the Four-Agents-Decoction (FAD) group at treatment cycle 1, and the other was from the placebo group at treatment cycle 2.

### Recruitment

Table 1 shows the time periods of recruitment, washout, baseline, treatment, and follow-up phases. The trial took a total of eighteen and a half months from January 2, 2003 to July 16, 2004.

**Table 1:** Time periods of trial progression.

Phase	Activity	Dates (m/d/year)
Recruitment	Assessment for eligibility	01/02/03∼5/26/03
Wash-out	Informed consent collection Medical screening visits	06/02/03∼09/17/03
Baseline	Pre-treatment clinic visits	11/20/03∼12/18/03
Treatment	Cycle 1–4 clinic visits	12/12/03∼04/15/04
Follow-up	Cycle 1–3 clinic visits Post-treatment examination	03/18/04∼07/16/04

### Baseline data

The women in the two groups were comparable in age, college education, weight, body-mass index, menstruation duration, and cycle length (Table 2). Experience with the product in the two groups was similar and no further adjustment was to be made in the outcome analysis.

**Table 2:** Pre-randomization characteristics of the 78 women with primary dysmenorrhoea.

Characteristic	Four-Agents-Decoction(n = 39)	Placebo (n = 39)
Age mean (SD*), years	23.6 (2.97)	23.3 (2.92)
College education, n (%)	39 (100)	39 (100)
Past user or believer, n (%)	39 (79)	39 (76)
Weight mean (SD), kg	51.7 (5.71)	52.0 (6.01)
Body-mass index mean (SD), kg/m^2^	19.8 (2.27)	19.8 (1.75)
Menstruation duration mean (SD), days	5.66 (0.98)	5.77 (0.95)
Menstrual cycle length mean (SD), days	27.2 (2.76)	27.2 (3.52)

* SD, standard deviation

During wash-out and baseline phases the reported symptoms episodes of symptoms-inner heat reaction, abnormal menses, PMS-like symptoms, respiratory disorder or illness, and gastrointestinal disorder were few but slightly higher in the 39 participants in the FAD group for a total of 147 cycles than in the 39 participants in the placebo group for a total of 152 cycles (Table 3).

**Table 3:** Self-reported episode frequency (person-episodes) of symptoms described in traditional Chinese medicine during the pre-treatment (wash-out and baseline cycles) and treatment phases.

Symptom	Four-Agents-Decoction		Placebo	
	Pre-treatment (n = 39) (147 cycles)	Treatment (n = 38) (134 cycles)	Pre-treatment (n = 39) (152 cycles)	Treatment (n = 39) (137 cycles)
Inner heat reaction	6	7	1	5
Abnormal menses	5	6	3	2
PMS-like	46	38	29	22
Respiratory disorder or illness	24	34	20	20
Gastrointestinal disorder or illness	11	12	12	8

### Numbers analyzed

Two women failed to comply with the treatment, resulting in 97.4% compliance rate. The numbers of participants at baseline and the treatment phase from cycle 1 to 4 were 39 and 38, 38, 38, 20 for the Four-Agents-Decoction (Si Wu Tang) group and 39 and 39, 38, 38, 22 for the placebo group.

### Outcomes and estimation

**Figure 2:**
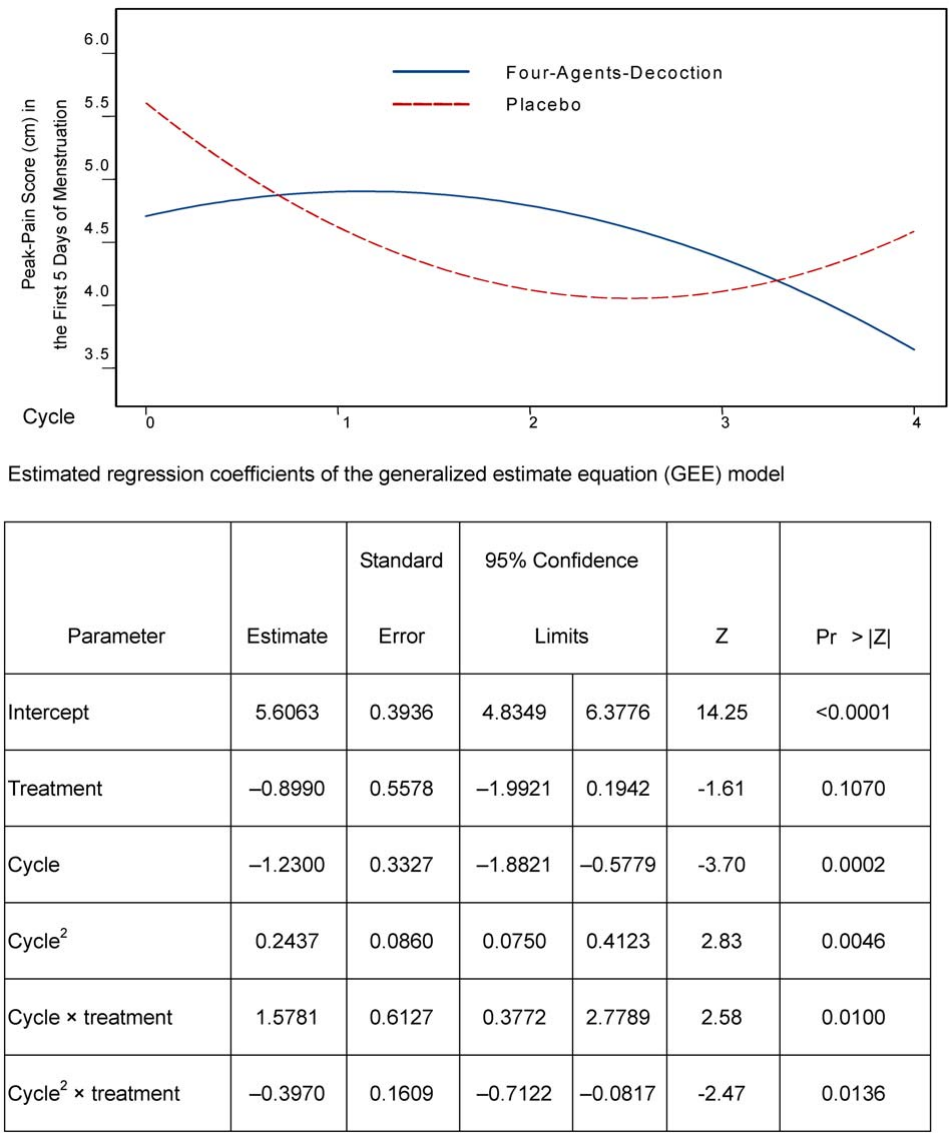
Peak-pain change based on the GEE model. Y-axis: Peak-Pain Score (cm) in the First Five Days of Menstruation. X-axis: Cycle (0 is the baseline cycle closest to the start of treatment, 1–4 are treatment).

Visual analog pain intensity was our outcome. The GEE model (Figure 2) fitted with the explanatory variables, *treatment and cycle*, by the approach with “unstructured working correlation” [21], shows trends of peak-pain decreasing in the FAD group and increasing in the placebo group. Regression parameter estimates along with the robust estimates of standard errors and test significance are detailed in the table following the graph. The model indicates a highly significant treatment effect, which was explained by the interaction terms- cycle*treatment (*p* = .010) and cycle^2^*treatment (*p* = .0136)- after the difference in the peak-pain scores of the two groups was adjusted for cycle trends.

**Figure 3:**
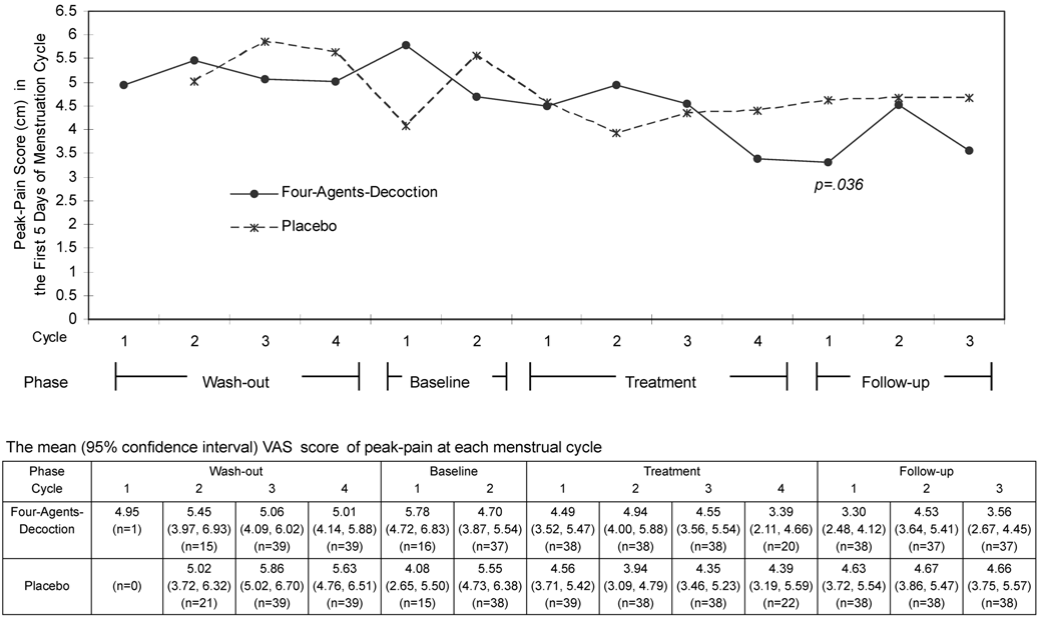
Distribution of peak-pain intensity throughout the trial. Y-axis: Peak-Pain Score (cm) in the First Five Days of Menstruation. X-axis: Cycles within the phase. Phases of the entire trial.

**Figure 4:**
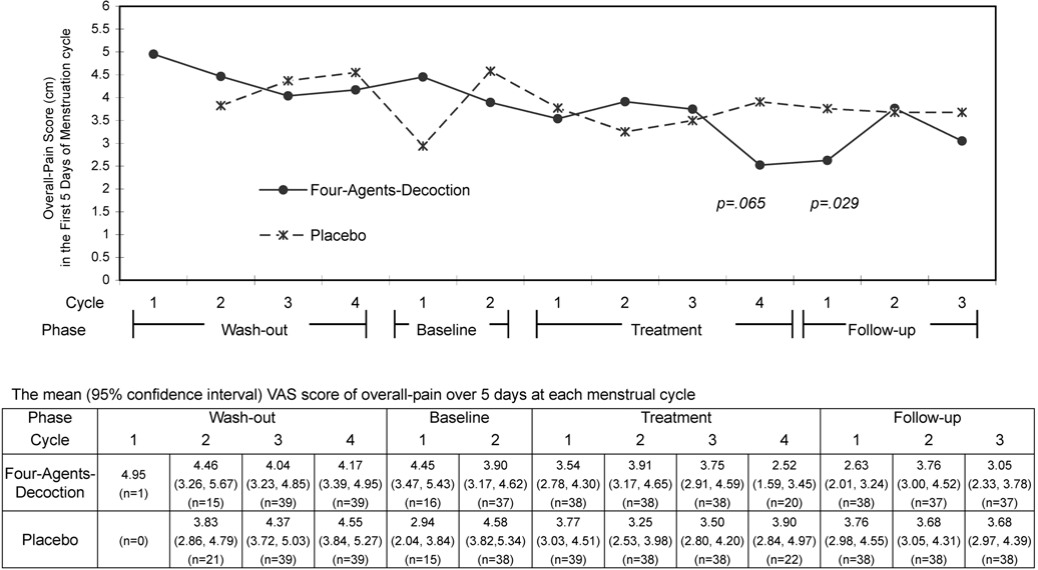
Distribution of overall-pain intensity throughout the trial. Y-axis: Overall-Pain Score (cm) in the First Five Days of Menstruation. X-axis: Cycles within the phase. Phases of the entire trial.

These 78 women's menstrual pain pattern throughout the trial is shown in Figure 3 for their peak-pain intensity and in Figure 4 for the over-all pain intensity. The two distribution patterns were similar. The mean and 95% CI of pain scores are shown along with the line graphs. The pain of both groups diminished after treatment cycle 1. However, at treatment cycle 2, the pain increased in the active group, whereas the pain was the least in the placebo group. At treatment cycle 3, the pain intensity was decreased in the FAD group and increased in the placebo group; the changes persisted to the treatment cycle 4 and follow-up cycle 1. With a rise at follow-up cycle 2 and 3, the pain scores of the FAD group were still lower than those during the pre-treatment phase. On the other hand, the scores of the placebo group remained similar over the post-treatment phase. The two treatment groups differed at treatment cycle 4 insignificantly; but the difference reached statistical significance at follow-up cycle 1 (*p* = .029 for overall-pain and *p* = .036 for peak-pain).

**Figure 5:**
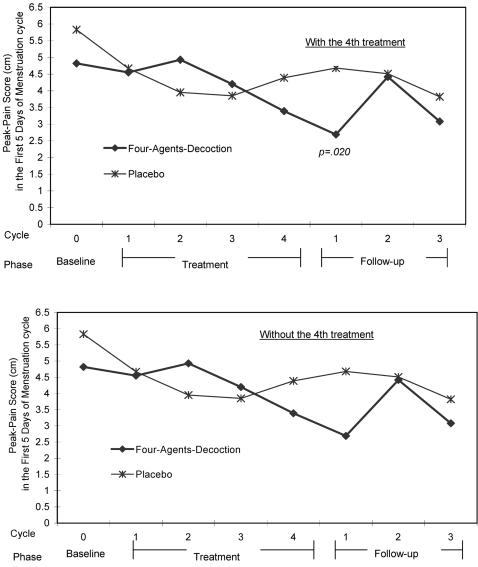
Peak-pain intensity distribution stratified by whether women had the 4th treatment cycle. Y-axis: Peak-Pain Score (cm) in the First Five Days of Menstruation. X-axis: Cycles within the phase. Phases of the entire trial.

Peak-pain intensity was further analysed. The mean reduced peak-pain score at follow-up cycle 1 in the FAD group did not differ significantly by treatment length (*p* = .125). Figure 5 shows the reduced peak-pain did differ profoundly among women treated for four menstrual cycles (2.69 (2.06) cm, mean (standard deviation), for the 20 women with FAD and 4.68 (3.16) cm for the 22 women with placebo, *p* = .020.) The reduction was more than 2 cm in the FAD group, whereas less than 1 cm in the placebo group. The GEE model also indicated a significant beneficial treatment effect in these 42 women.

The consumption of ibuprofen and other analgesics was low during the pre-treatment phase, as well as in the subsequent treatment cycles. Of the women who ever took study-provided ibuprofen during a menstrual period, the percentage of women using one tablet decreased from baseline to treatment cycle 1–4, 43.6% to 31.6%, 28.9%, 21.1%, and 25.0% for the FAD group and 23.1% to 15.4%, 21.1%, 21.1%, and 18.2% for the placebo group. And using more than one tablet was 12.8%, 10.5%, 21.1%, 28.9%, and 10.0% for the FAD group, and 28.2%, 20.5%, 13.2%, 13.2%, and 18.2% for the placebo group, respectively. For the use of other analgesics, including ibuprofens of other brands and acetaminophens, the percentage was very low, 0%, 5.26%, 5.26%, 2.63%, and 0% for the FAD group and 0%, 5.13%, 0%, 2.63%, and 2.63% for the placebo group. Twenty-four women in the FAD group and 19 women in the placebo group in the pre-treatment phase had ever used other analgesics or additional remedies, including heating treatment, drinking hot liquids, eating chocolates or black sugar, or physical exercise, etc., the number of users decreased in both groups during the treatment phase; 18 women in the FAD group and 14 women in the placebo group.

We did not obtain significant numbers of dichotomous responses (pain or no pain) during or at the end of treatment phase when the FAD and placebo groups were compared. The percentage of women having no menstrual pain in the FAD group was slightly higher during treatment cycle 1–4, 13.2%, 10.5%, 10.5%, and 15% compared with 2.6%, 7.9%, 5.3%, and 9.1% in the placebo group, respectively (Table 4). The study was not powered to assess the outcome of work or school absence; four women from the FAD group and two from the placebo group took absence from work or school during the treatment phase.

**Table 4:** Percentage of women who did not feel pain after receiving the treatment.

Treatment	Four-Agents-Decoction % (n)	Placebo % (n)
Cycle 1	13.2 (5/38)	2.6 (1/39)
Cycle 2	10.5 (4/38)	7.9 (3/38)
Cycle 3	10.5 (4/38)	5.3 (2/38)
Cycle 4	15.0 (3/20)	9.1 (2/22)

### Ancillary analyses

At the end of treatment, 92% (35/38) of the FAD group and 53% (20/38) of the placebo group correctly identified the content of their capsules. Of the women who guessed correctly, 62.9% (22/35) and 20% (4/20) in the respective groups had gastric reflux (belch) during the course of treatment. Gastric reflux occurred more often with FAD but had no association with the treatment length of three or four cycles. Seventy-five percent (12/16) of women with FAD and that guessed their group correctly when surveyed at the end of 3-cycle treatment had gastric reflux, compared to 33.3% (2/6) in the placebo group. The gastric reflux occurred less in women who had four treatment cycles and guessed their group correctly, 52.6% (10/19) women with FAD and 14.3% (2/14) women with placebo. Only one woman in the FAD group reported in the post-trial questionnaire that her reported pain intensities were affected by the released aroma.

Pulsatility index of uterine arteries (PI) was used as a complementary tool to evaluate menstrual pain [24]. It is an averaged value of the measurements taken by both gynaecologists in alternate order on each participant during her menstruation at ultrasound examinations for pelvic lesions. Both PI (pulsatility index) of right and left uterine arteries decreased gradually from day 1 to day 5 at both screening and post-treatment (Table S1), the trend corresponded with the pattern of VAS pain scores.

### Adverse events

Reactions associated with FAD noted by traditional Chinese medicine physicians were inner heat reaction, such as blisters and pimples, and disturbed menstruation, such as amenorrhea and menorrhagia. The mean weight (standard deviation) of soiled pads was not different between pre-treatment and treatment phases, 68.8 (37.2) and 70.4 (35.0) g for the FAD group, and 75.8 (44.3) and 75.0 (49.3) g for the placebo group. The numbers of self-reported symptom episodes after the treatment were still small, but changed slightly. The most notable change was respiratory disorder, which increased 10 episodes in the FAD group but none in the placebo group. Both groups had a decreased episode of PMS-like symptoms, 8 in the FAD and 7 in the placebo group. The increase in the FAD group was only one episode for inner heat reaction, abnormal menses, or gastrointestinal disorder or illness (Table 3).

No levels of blood chemicals changed, except for an increase in heavy metals at the end of treatment. This slight increase occurred not only in the FAD group but also in the placebo group (Table 5).

**Table 5:** Mean (standard deviation) haematological parameters at screening (the last wash-out cycle) and post-treatment (end of treatment) of the 76 women.

Biochemical	Four-Agents-Decoction		Placebo	
	Screening (n = 38)	Post-treatment (n = 38)	Screening (n = 38)	Post-treatment (n = 38)
CA 125, U/mL	17.9 (7.42)	17.0 (9.85)	17.9 (6.63)	16.9 (9.08)
AST, IU/L	18.6 (4.06)	18.3 (3.31)	18.1 (3.76)	19.2 (4.62)
ALT, IU/L	13.2 (5.72)	13.7 (4.78)	12.3 (5.75)	12.4 (4.52)
Uric acid, mg/dL	4.40 (1.32)	4.17 (1.06)	4.40 (1.15)	4.28 (0.97)
Urea nitrogen, mg/dL	10.5 (2.29)	10.9 (2.85)	11.2 (2.31)	11.2 (2.87)
Creatinine, mg/dL	0.79 (0.11)	0.77 (0.08)	0.79 (0.09)	0.80 (0.08)
Lead, µg/L	1.82 (0.78)	2.47 (1.06)	1.39 (0.48)	2.61 (1.06)
Mercury, µg/L	2.29 (1.71)	3.94 (1.72)	3.06 (2.42)	3.71 (1.33)
Arsenic, µg/L	6.89 (3.11)	8.21 (2.94)	7.29 (4.75)	8.36 (3.86)
Copper, µg/L	955.3 (116.0)	994.7 (135.8)	990 (151.9)	1037 (149.7)
Cadmium, µg/L	0.99 (0.38)	1.03 (0.50)	0.94 (0.43)	0.96 (0.50)
Serum Iron, µg/dL	66.1 (37.2)	64.6 (32.8)	74.7 (31.5)	66.0 (30.4)
Hemoglobin, g/dL	12.6 (0.89)	12.4 (1.00)	12.6 (0.85)	12.6 (0.96)
Hematocrit, %	38.8 (2.32)	37.0 (2.42)	38.6 (2.08)	37.3 (2.34)

Regulated limits and actual levels of heavy metals in the study product-FAD: lead<20 ppm, 0.45 ppm; arsenic<5 ppm, 0.14 ppm; cadmium<0.5 ppm, 0.01 ppm; mercury<0.5 ppm, 0.02 ppm.

## Discussion

### Interpretation

Menstrual pain is the major symptom of primary dysmenorrhoea. Both the overall-pain and peak-pain improved, after three cycles of FAD treatment and persisted to the follow-up phase. At follow-up cycle 1, the peak-pain score in the FAD group decreased 2.14 cm and 33.9%, meeting the minimum requirement of 1.3 cm and 33%, respectively, for clinical significance with respect to a change in pain severity [25]–[26]. As menstrual pain on the most intense day is considered unbearable for many dysmenorrheic women, this appears to be a delayed treatment benefit of FAD.

The methodology used to conduct a clinical trial of herbal remedies is the foremost criterion before efficacy is assessed [27]. To the best of our knowledge, this study is the first trial of a documented traditional Chinese herbal formula conducted as rigorously as those for synthetic drugs. First, we randomised a group of young women that were diagnosed concurrently by two conventional Western physicians to differentiate primary from secondary dysmenorrhoea.

Second, both conventional Western and traditional Chinese medicine physicians participated together in the same trial to observe possible adverse reactions or events from FAD. Our observation of an increase in episodes of respiratory illness was unexpected and might be due to chance, as the treatment phase fell during the winter, when such illnesses increase greatly among the general public. We do not rule out the need of scrutiny in future trial. Adverse reactions or events historically attributed to traditional Chinese medicine, such as inner heat reaction or disturbed menstruation, did not increase in the FAD group compared with the placebo group. There were no abnormalities of hepatic or renal function, or heavy metal toxicity attributable to the FAD as compared to placebo. Our regimen for this formula appears safe.

Third, we masked the distinctive aroma of the FAD. Although we validated our double-blind approach before recruitment, the high rate of the women identifying the study product correctly from unavoidable gastric reflux in the FAD group may be attributed to the large dose or familiarity with this commonly used herb formula [28]. However, we took several approaches to allow the best possible assessment for the pain. Specifically, (a) we stratified the women by their experience with the formula before the randomisation; (b) in order to control the quality of subjective self-reported pain score data, we collected daily pain reports using an unmarked VAS scale in diaries on our Web site, in an effort to prevent them from making day by day comparison and changing their instant subjective pain feeling; and (c) we compensated the self-reported subjective measure by using PI from the pelvic ultrasonography to serve as a complementary measure. The trend of PI at the screening phase, while the PI and VAS pain score were not affected by the treatment, is consistent with the reports that a higher PI is related to more severe primary dysmenorrhoea [29]–[30], and both PI and VAS pain score are the highest in the first day of menstruation cycle [30]. Our self-reported VAS pain score has been reliable for evaluating the effectiveness.

Despite the improvement on methodology for conducting a rigorous clinical trial of traditional Chinese herbal medicine, this study had limitations on recruitment, as very few sufferers can be found at regular clinics. Although we received high response to our recruitment questionnaire from the community, the low participation-fewer than 50% (from 215 to 102 women) of the initially screened respondents made their first hospital visit for the trial information session and clinical evaluation-could be due in part to the SARS outbreak. Owing to the time strain, we went on with the 102 participants to be confirmed by the study gynaecologists for primary dysmenorrhoea. Ninety-five primary dysmenorrheic women passed the clinical screening and 78 women entered the treatment. The 78 treated participants exceeded the minimum sample size of 56 but less than the anticipated number of 100.

Operation of ad hoc study clinic visits by group may have reduced the variability on the diagnosis by different physicians, the drop-out rate, and the time to complete the entire trial, but variations inherent in menstruation are unavoidable, which require greater manpower to closely follow up the women in order to meet the clinic schedules. Menstrual cycle length in the FAD and the placebo groups was similar in both pre-randomization and treatment phases, but mean (standard deviation) changed from 27.2 (2.76) to 32.1 (5.08) days and from 27.2 (3.52) to 31.4 (4.09) days, respectively, resulting in an extra cycle of baseline for 31 women and an extension of treatment from three to four cycles for 42 women.

### Generalizability

Whether FAD has a clear curing effect for use in clinical medicine to treat primary dysmenorrhoea needs to be elucidated by a larger trial. It will require detailed sample size calculation covering different geographical areas, age groups, and ethnic groups or races for assessing generalisability. A longer treatment phase should be incorporated for obtaining optimal dose and treatment length. Multiple batches of the product can also be included for determining the extent of the efficacy.

### Overall evidence

We did not find the same distinct effect from FAD treatment among 78 women as from synthetic analgesics or a significant reduction of menstrual pain after a pre-determined three-month (three cycles) study period of treatment [12]. But a larger clinical trial with 260 participants may obtain significant pain improvement by a formula composed of three Chinese herbs [31].

Starting from the treatment cycle 3, both the overall-pain and the peak-pain intensity reduced in the FAD group and rose in the placebo group. The changes from 4.82 at baseline to 3.30 cm at follow-up cycle 1 for the FAD group and from 5.51 to 4.63 for the placebo group are comparable to the study of vitamin E treatment for dysmenorrhoea, in which the pain reduction was from 5.5 to 3.5 with vitamin E versus 5.4 to 4.3 with placebo after two months of treatment [19]. The persistent pain reducing effect at treatment cycle 4 and follow-up 1 indicates that a longer treatment and follow-up is required in future trials so that the slow releasing characteristic inherent in the theory of traditional Chinese medicine can be accurately assessed.

To the best of our knowledge, no active ingredients in FAD have been identified. In our post hoc preliminary study of the mechanism of action of Four-Agents-Decoction for treating primary dysmenorrhoea, cyclooxygenase-2 (COX-2) was inhibited by 85% in 1% of the same solution that was used for assessing quality control of the study product. Our series of studies from the historical use, the surveys of usages, the post-market clinical trial, to the biochemical mechanism provide the clinical plausibility for FAD in relieving the worst menstrual pain intensity unrelated to organic lesions.

## Supporting Information

Figure S1 Custom-made study product: Four-Agents-Decoction (Si Wu Tang)
(2.81 MB TIF)

Table S1 Distribution of pulsatility index and the corresponding pain score on visual analog, presented as mean (standard deviation).
(0.03 MB DOC)

Protocol S1 Trial Protocol
(0.43 MB PDF)

Checklist S1 CONSORT Checklist
(0.11 MB DOC)
