# Supplementary figures and images for: A Randomised Placebo-Controlled Trial of a Traditional Chinese Herbal Formula in the Treatment of Primary Dysmenorrhoea
Source: PLoS One. 2007 Aug 15;2(8):e719. doi: 10.1371/journal.pone.0000719 (PMC1940310; doi:10.1371/journal.pone.0000719)

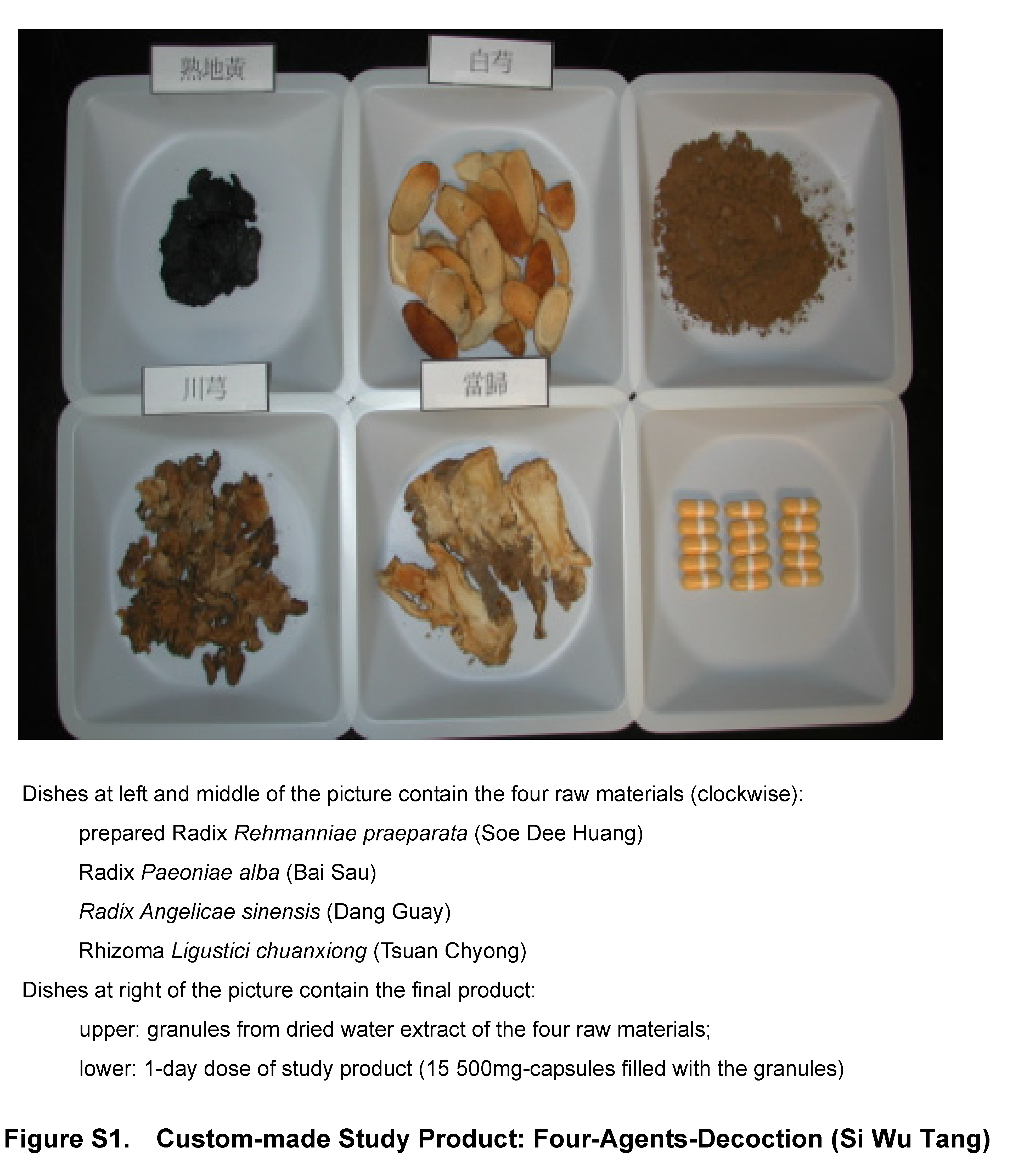

Supplement: Figure S1 — Custom-made study product: Four-Agents-Decoction (Si Wu Tang) (2.81 MB TIF) [file pone.0000719.s001.tif]
