# Supplementary material for: A Randomised Placebo-Controlled Trial of a Traditional Chinese Herbal Formula in the Treatment of Primary Dysmenorrhoea
Source: PLoS One. 2007 Aug 15;2(8):e719. doi: 10.1371/journal.pone.0000719 (PMC1940310; doi:10.1371/journal.pone.0000719)
Supplement: Table S1 — Distribution of pulsatility index and the corresponding pain score on visual analog, presented as mean (standard deviation). (0.03 MB DOC) [file pone.0000719.s002.doc]

Table S1. Distribution of pulsatility index and the corresponding pain score on visual analog, presented as mean (standard deviation)

| Examination Phase | Measure | 3 days prior to menses | Menses with diary record | | | | | No menses |
| --- | --- | --- | --- | --- | --- | --- | --- | --- |
|  |  | No diary | Day 1 | Day 2 | Day 3 | Day 4 | Day 5 | Day 6 |
| Screening  (N=78) | Left pulsatility index* | 2.99 (0.01)  (n=2) | 4.09 (1.02) (n=17) | 4.04 (0.86) (n=18) | 3.66 (0.79) (n=20) | 3.38 (0.70) (n=14) | 3.42 (0.65) (n=6) | 3.74  (n=1) |
|  | Right pulsatility index | 2.98 (0.16)  (n=2) | 4.10 (1.11) (n=17) | 3.89 (0.81) (n=18) | 3.71 (0.84) (n=20) | 3.48 (0.84) (n=14) | 3.11 (0.62) (n=6) | 3.45  (n=1) |
|  | Pain score of the day (cm) |  | 4.77 (2.64) (n=17) | 3.27 (2.78) (n=18) | 1.24 (2.65) (n=20) | 0.39 (1.31) (n=14) | 0  (n=6) |  |
| Post- treatment  (N=75) | Left pulsatility index |  | 3.65 (0.67) (n=11) | 3.77 (0.63) (n=25) | 3.36 (0.99) (n=20) | 3.14 (0.66) (n=15) | 3.27 (1.06) (n=4) |  |
|  | Right pulsatility index |  | 3.59 (0.73) (n=11) | 3.79 (0.67) (n=25) | 3.39 (1.01) (n=20) | 3.07 (0.55) (n=15) | 3.35 (1.37) (n=4) |  |
|  | Pain score of the day (cm) |  | 3.96 (2.87) (n=11) | 2.84 (2.98) (n=25) | 1.96 (2.93) (n=20) | 0.46 (1.77) (n=15) | 0  (n=4) |  |

*, Pulsatility index: (maximal systolic flow-minimal diastolic flow)/mean flow
