# Supplementary material for: A Randomised Placebo-Controlled Trial of a Traditional Chinese Herbal Formula in the Treatment of Primary Dysmenorrhoea
Source: PLoS One. 2007 Aug 15;2(8):e719. doi: 10.1371/journal.pone.0000719 (PMC1940310; doi:10.1371/journal.pone.0000719)
Supplement: Protocol S1 — Trial Protocol (0.43 MB PDF) [file pone.0000719.s003.pdf]

Text S2. Trial protocol

國家衛生研究院人體試驗醫學倫理委員會
研究計劃書

■藥品 □醫療器材 □醫療技術 □其他_____

一、計畫名稱：

中文：以隨機雙盲臨床試驗評估四物湯膠囊治療經痛的療效

英文：Intervention Trial of Four-Agents-Decoction for Dysmenorrhea in Young College Women

二、主要主持人：

葉梁蘭蘭博士

三、協同主持人：

蔡德豐醫師、劉裕森醫師、王麗香醫師、劉嘉燿醫師、張恆鴻醫師

四、試驗主題：

Finding effective traditional Chinese herbal medicine has now become one of the interests of medicine, pharmacology, and agricultural biotechnology worldwide. Among the thousands of herbals, some herbs have been freely used among Chinese. Formula or prescriptions listed in the ancient pharmacopoeia of traditional Chinese medicine are usually composed by 3-5 herbs for treatment of illnesses. However, there are no consistent and systematic evidence-based data to support their efficacy or precise biochemical analysis of their complicated compositions. This is partly due to the lack of uniformity of the source of the herbs, the method of their production, and the method of preparation of the concoctions. The gold standard of obtaining evidence for efficacy is to conduct a randomized double-blind placebo-controlled clinical trial. However, the problem arises about the selection of safe herbs or prescriptions to be tested in human experiments. The survey of the prevalence of the use of Chinese medicines among the employees of a research institution in August 2000 found that four-agents-decoction was ranked first among the named Chinese medicine that had been most frequently used in the previous year and the usage was for prophylaxis and relief of menstrual pain. Most of the users obtained the materials without consulting traditional Chinese medicine practitioners. No reports of adverse reactions or warning in use of four-agents-decoction have ever been published; either did the users report in this survey.

Four-agents-decoction consists of four herbs, prepared Radix Rehmanniae praeparata (熟

地黃), Radix Angelicae Sinensis (當歸), Radix Paeoniae Alba (白芍), and Rhizoma Ligustici Chuanxiong (川芎), which has been used as a basic formula in traditional Chinese medicine since Sung Dynasty. Women's illnesses are among the most commonly treated with traditional Chinese medicine. A few clinical studies conducted in other countries have tested or are testing similar herbs or formulas containing herbs present in four-agents-decoction for the relief of dysmenorrhea. Four-agents-decoction can thus be considered a safe formula; there should not be any delay of conducting a randomized double-blind placebo-controlled parallel intervention trial of four-agents-decoction for dysmenorrhea in Taiwan.

This is a pragmatically oriented pilot trial incorporated with an observational epidemiologic study. Subjects are healthy young college women who have suffered from primary dysmenorrhea for at least four consecutive times for the past six cycles will be recruited from Chinese Institute of Technology and Takming College. The research activities include recruiting, randomizing, washing-out, establishing baseline, treating subjects with four-agents-decoction and placebo for three cycles, and following-up three cycles for the assessment of menstrual pain. Examinations for screening and monthly check up are at the Taipei Municipal Chung Hsiao Hospital for women from Chinese Institute of Technology and at the Tri-Service General Hospital for women from Takming College. At the start and end of the trial, five traditional Chinese medicine practitioners will examine signs and symptoms independently and concurrently on each of the subjects using a standardized checklist. The scores of the symptoms and signs will be further evaluated for the validity of differentiated patterns in determination of the efficacy of four-agents-decoction.

This intervention trial (FADD) will be conducted in collaboration with other nine specialists, including three gynecologists, four traditional Chinese medicine physicians, one pharmacologist, and one statistician. An estimate of 15 months is needed to complete this trial.

五、試驗目的及背景說明（包括學理根據及有關文獻報告）：

Four-agents-decoction, containing prepared Radix Rehmanniae praeparata (熟地黃), Radix Angelicae Sinensis (當歸), Radix Paeoniae Alba (白芍), and Rhizoma Ligustici Chuanxiong (川芎), is an ancient formula in the treatment of women illnesses since Sung Dynesty. This theory was recently emphasized in Taiwan (1), mainland China (2), and Japan(3). A small scale clinical study conducted in mainland China (4) has shown the effectiveness of four-agents-decoction for prophylaxes or treatment of menstrual disorder. Of the 40 women between age of 14 and 50 who met the criteria of dysmehorrhea, 24 women (60%) after used the prescription had normal menstrual length and amount or the pain stopped. Another 12 women (30%) were improved to some degrees or had less pain. There was no recurrence of pain even three months after the treatment. Although no specific warnings in use of this aged formula were ever reported (5), the efficacy of this formula for treating dysmenorrhea has never been studied in a randomized, double-blind placebo-controlled clinical trial.

Working women in Taiwan can be granted one sick day off each month due to dysmenorrhea. In a sample of college women the prevalence rate of dysmenorrhea was 82% (unpublished data); the pain intensity ranged from slight pain, pain, to severe pain on four-point pain scale. The morbidity of dysmenorrhea had been hidden and private, but now it becomes a challenge to public health. While the effectiveness of oral contraceptives or analgesics for relieving menstrual pain is evident in general practice of Western medicine,

there is a subset of women who do not respond to this type of conventional treatment and seek althernative medicine or therapy (6) . A variety of basic formulas with substracted and added materials (加減方) and fixed materials in single formulas (固定方) has been available in traditional Chinese medicine (7), in which prepared Radix Rehmanniae Praeparata (熟地黃), Radix Angelicae Sinensis (當歸), Radix Paeoniae Alba (白芍), and Rhizoma Ligustici Chuanxiong (川芎) are the major materials in 40 of the formulas (8). Starting a week prior to and until the onset of menstruation as a preventive measure has been suggested for an effective relief of the pain (9-10). Over 60% of dysmenoric students in the Chinese Technology Insitute have used this remedy in a variety of forms for prophylaxis to menstrual discomfort (unpublished data). Four-agents-decoction in powder or tablets is reimbursed by the National Health Insurance Bureau. However, the efficacy of this formula has never been evaluated, either has the dosage. Knowledge of the efficacy based on clinical evidence will be beneficial for women, employers, and National Health Insurance Bureau.

A formula similar to four-agents-decoction, Toki-shakuyaku-san, which contains three of the four herbs consisted in four-agents-decoction, also shown similar effectiveness in the treatment of dysmenorrhea among 40 subjects who were equally divided into two groups (11). The dose of the medicine in this double-blind clinical trial was 7.5 g/day, taken three times daily. The trial took a period of 6 menstrual cycles, including 2 cycles each of wash-out, treatment, and follow-up. The subjects who had suffered from primary dysmenorrhea for one or more years, and who had a combination of cold, deficiency, yin, and blood stagnation, were selected at the recruitment according to the diagnostic scoring system derived in Japan. Both pain intensity and the consumed amount of rescue analgesics were decreased over the two treatment cycles and significantly lower in the treatment group compared to the placebo group. These differences persisted during the two follow-up cycles after cessation of the treatment.

The cause of primary dysmenorrhea proposed in conventional Western medicine is ischemia in uterine by increased contractility and decreased blood flow from the excessive production of prostaglandins. In traditional Chinese medicine, menstrual pain is thought due to inhibition of qi and blood stasis. The level of $PGF_2\alpha$ in both peripherial and menstrual blood of dysmenorrheic women was lowered by 21.6% and 38.7%, respectively, after the treatment with Powder of Angelicae Sinensis and Paioniae (當歸芍藥散); whereas no difference was found between pre- and post-treatment in controls (12). While the mechanism of four-agents-decoction in relieving menstraul pain is not clearly known, four-agents-decoction has been found with high nutrition values of Fe, Zn, Mn, Co, Cd and folic acid, as well as amino acids, such as aspartic acid and arginine (13-15).

Theory of indivisability between Chinese medicine（中醫）and Chinese medicine prescription or formula（中藥）is the fundermental of traditional Chinese medicine; however, the diagnostic system has been mysterious and is considered inaccurate to Western medicine practitioners. A recent study (16) conducted in the US has found that the degree of consistency between diagnoses made by nine practitioners in US, who had been trained at different places, was high only for kidney yin deficiency in 23 women with menopausal symptoms. The high concordance for kidney yin deficiency could be due to high frequency of the sign that is defined broadly as a major sign in differentiated diagnosis; whereas the concordance for other signs or symptoms, which are more complex by the theory of traditional Chinese medicine, was somewhat low. These findings indicate considerable

variations exist among practitioners. For menstrual pain, at least seven differentiated pattern systems have been practiced by traditional Chinese medicine practitioners in treatment of dysmenorrhea (17). The inconsistency and variations are often perceived as a result of practitioners' subjective four examiniations (四診): inspection (望診), listening and smelling (聞診), inquiry (問診), and palpation (切診) in determining patterns from differentiated diagnoses,.

Two versions of diagnosis for menstrual pain (18-19) have been officially instituted and are presently practiced in mainland China; no specific guidelines have been published for people living in Taiwan. The scoring systems used in the previously stated study (11) has changed the descriptive qualitative diagnoses of signs and systems into quantitative measures to make the evaluation towards consistency among practitioners and in turn provides more specific diagnosis. Through careful examinations and discussions among a group of traditional Chinese medicine physicians after analyzed by using epidemiologic methods, the differented patterns of individuals may well be determined by scoring symptoms and signs. This diagnosis system for primary dysmenorrhea can be further developed for its entry into clinical practice in Taiwan.

六、試驗方法：

(一)受試者選擇標準（Patient eligibility）

(1) inclusion criteria

Women aged 18 years and older are currently enrolled in China Technology Institute and Takming College, and meet the following criteria:

(a) had regular periods (26-33 days) in the past 6 months
(b) experienced at least 4 consecutive painful periods
(c) pain started one day before or on the onset of bleeding
(d) taken analgesics

(2) exclusion criteria

(a) began experiencing menstrual pain at menarche
(b) started having pain more than 3 years after menarche
(c) took oral contraceptive pills
(d) had sexual life
(e) had endometriosis; pelvic inflammatory disease; inflammatory bowel syndromes
(f) had gynecological surgery, including pregnancy

(二)試驗設計與流程（與人體試驗相關部份）：

The study flow is shown in Appendix 1.

A. Recruitment of Subjects

1. **Survey---**identification and selection for primary dysmenorrheic women

Administering a questionnaire (Appendix 2) to survey 400 women who have experienced menstrual pain in the past six months at China Technology Institute and Takming College. Approval for conducting the survey has been granted by the IRB of

NHRI (Appendix 3). The questionnaire will be sent to 400 women by mail. The women can either send the questionnaire back to NHRI or hand it to the nursing teacher, 郝冬梅 or 謝伶俐. The gift, tote bag, will then be given out by the teacher. The data in the questionnaires will be analyzed and examined by the criteria set for this trial. Among those met the criteria listed below (IV-A-2), a total of 150 women initially eligible for the trial are to be recruited.

An informed consent (Appendix 4) will be sent to these 150 women for preview. Later a meeting will be held for these women and their parents or guardians, in which the purposes of the trial will be explained and the procedures of this trial including guidelines of filling out questionnaires, diary and menstrual record will be gone over, and any questions in the content of informed consent will be answered. A signed informed consent will be collected from those interested to participate at the end of meeting. (The contents of the meeting and handbook will be focused on explanation of the trial's characteristics and significance, which will be planned and given by two young physicians, the Co-investigators.) Motrin for 3 days will be given to the participants, which is the only medication and method to be used in wash-out period. These women will then be confirmed for primary dysmenorrhea with ultrasound by gynecologists at Taipei Municipal Chung Hsiao Hospital and Tri-Service General Hospital.

2. **Criteria for pre-screening subjects** (Appendix 5)

(1) inclusion criteria

Women aged 18 years and older are currently enrolled in China Technology Institute and Takming College, and meet the following criteria:

(d) had regular periods (26-33 days) in the past 6 months
(e) experienced at least 4 consecutive painful periods
(f) pain started one day before or on the onset of bleeding
(d) taken analgesics

(2) exclusion criteria

(g) began experiencing menstrual pain at menarche
(h) started having pain more than 3 years after menarche
(i) took oral contraceptive pills
(j) had sexual life
(k) had endometriosis; pelvic inflammatory disease; inflammatory bowel syndromes
(l) had gynecological surgery, including pregnancy

3. **Arrangement of Clinic visit**

Clinics close to the two colleges are selected. Fifty CTI students go to Taipei Municipal Chung Hsiao Hospital (CHH) and 100 TMC students go to Tri-Service General Hospital (TSGH).

(1) All women will be traced attentively for menstrual cycles by study nurse 賴美齡, the women will be asked to visit hospitals between the 7th and 12th days of cycle.
(2) Clinic visits are arranged on one designated morning or afternoon specifically for the study.

(3) Study nurse 賴美齡 will be attending at both hospitals.
(4) All examiners from both hospitals, including study nurse, gynecologists, traditional Chinese medicine physicians, will have regular meetings led by Dr. JY Liu for setting up standardization of procedures, discussing unclear details, and trial operations at clinics. All the contents will be documented as parts of the protocol.
(5) All forms and necessary stationary supplies will be furnished by the study center located in 710, Nan-Kang Campus of NHRI.
(6) Dr. TW Liu, a consultant of this trial, will provide his experiences in conducting clinical trials to study physicians. He will provide trainings if necessary.

B. Wash-out—one full cycle (WV1)

1. Gynecology examination

Drs. 劉裕森 at CHH and 劉嘉燿 at TSGH

(a) performs check-up
(b) fills out the checklist of menstrual symptoms (Appendix 6)
(c) prescribes Motrin for 3 days for rescuing pain in next menstruation
(d) records all oral and written communications with other investigators and subjects
(e) supports care if emergency arises

2. Study nurse 賴美齡

(a) contacts subjects
(b) arranges clinic visits for subjects: registration, lead subjects to the clinic, etc.
(c) administers questionnaires (Appendix 8)
(d) gives out Motrin, copied menstrual record (Appendix 9), and diary (Appendix 10)
(e) takes information on emergency or any medical treatment for which women seek on their own
(f) makes copy of charts and stores at the study center
(g) checks the completion of procedures and records in the logs (Appendix 7)
(h) enters data into database

3. Nursing teachers, 郝冬梅 at CTI and 謝伶俐 at TMC

(a) gives out Motrin on gynecologist's order to subjects need extra
(b) traces students when necessary
(c) collects information on emergency or other medical treatment that subjects have sought on their own

C. Screening by Ultrasound Examination--Ancillary study (see XII)

1. Gynecology examination

Drs. 劉裕森 at CHH and 劉嘉燿 at TSGH

(a) performs ultrasound for the diagnosis of definite primary dysmenorrhea
(b) sends women home if not primary

(c) fills out the checklist of menstrual symptoms (Appendix 6)
(d) instructs women not to use any methods or medications before pain strikes
(e) prescribes Motrin for 3 days for next menstruation
(f) sends women to the next TCM clinic

2. Study nurse 賴美齡

(a) measures weight and height
(b) checks ultrasound record
(c) checks prescription
(d) administers questionnaires (Appendix 9)
(e) gives out Motrin, diary, and menstrual record form (Appendix 8)
(f) checks the completion of questionnaires administered by the 5 TCM
(g) makes copy of charts and stores in the study center
(h) checks the completion of gynecology visit and records in the logs (Appendix 7)
(i) enters data into database

After the confirmation, >100 subjects are enrolled.

D. Grouped Differentiated diagnosis--Ancillary study (see XII)

Drs. 陳建霖、張恒鴻、游智勝、蔡德豐、林高士

Women who have been confirmed for primary dysmenorrheic sufferers will receive differentiated diagnosis performed on by three of the five TCM physicians on Saturdays at Tri-service General Hospital. Each physician will be arranged to perform the diagnosis in similar frequency.

E. Baseline—one cycle (BV)

(For establishing baseline of pain intensity, some subjects may have to wait for one more cycle of wash-out.)

1. Gynecology examination

Drs. 劉裕森 at CHH and 劉嘉燿 at TSGH

(a) performs check-up
(b) fills out the checklist of menstrual symptoms (Appendix 6)
(c) prescribes Motrin for 3 days for rescuing pain in next menstruation
(d) records all oral and written communications with other investigators and subjects
(e) supports care if emergency arises

2. Laboratory examinations—all procedures are performed at the respective hospital and all supplies for the collection and shipping of blood specimens are provided by the hospital.

Clinic's Medical Technologists

(a) draw blood 35 ml (2 red, 2 green, and 1 purple top tubes) and perform the following analysis at respective hospital

i. CA-125 to exclude endometriosis
ii. Liver function and kidney function for the baseline of toxicity: GOT, GPT, BUN, creatinine, uric acid

iii. Anemic condition: hemoglobin, and hematocrit

(b) send 1 red top and 1 green top tubes to Taipei Institute of Pathology for testing toxicity of heavy metals: mercury, arsenic, lead, cadmium, and copper, and the test of iron and total binding protein capacity (TIBC)

3. TCM examination

Drs. 蔡德豐 at CHH and 王麗香 at TSGH

(a) diagnoses for differentiated patterns
(b) prescribes study products

4. Study nurse 賴美齡:

(a) contacts subjects, making sure the subjects are struck by menstrual pain
(b) arranges clinic visits for subjects: registration, lead subjects to the clinic, etc.
(c) excludes women having CA-125 > 35 or other abnormal values of biochemical parameters shown in the report
(d) administers questionnaires (Appendix 8)
(e) gives out Motrin, copied menstrual record (Appendix 9), and diary (Appendix 10)
(f) takes information on emergency or other medical treatment which women seek on their own
(g) makes copy of charts and stores at the study center
(h) performs centrifugation of whole blood and aliquots all leftover blood specimens, and brings back to store at study center
(i) checks the completion of procedures and records in the logs (Appendix 7)
(j) enters data into database
(k) delivers study products to the randomized subjects

5. Nursing teacher, 郝冬梅 at CTI and 謝伶俐 at TMC

(a) gives out Motrin on gynecologist's order to subjects need extra
(b) traces students when necessary
(c) collects information on emergency or other medical treatment that subjects have sought on their own

Women will be stratified by hospital into two strata and then randomized within each stratum to either treatment or placebo group using the method of block randomization (see VII B)

F. Intervention—3 cycles (IV1, IV2, IV3)

1. Gynecology examination

Drs. 劉裕森 at CHH and 劉嘉燿 at TSGH

(a) performs check-up
(b) fills out the checklist of menstrual symptoms (Appendix 6)
(c) prescribes Motrin for 3 days for rescuing pain in next menstruation
(d) records all oral and written communications with other investigators and subjects
(e) supports care if emergency arises

2. **TCM examination**

Drs. 蔡德豐 at CHH and 王麗香 at TSGH:

(a) diagnoses for differentiated patterns
(b) evaluates the use of study products
(c) prescribes study products for subsequent treatment

3. ***At the 3rd treatment cycle*, *another Laboratory Examination*--** all procedures are performed at the respective hospital and all supplies for collection and shipping of blood specimens are provided by the hospital.

Clinic's Medical Technologists:

(a) draw blood 35 ml (2 red, 2 green, and 1 purple top tubes) and perform the same analysis as at the baseline at respective hospital
   i. CA-125
   ii. Liver function and kidney function for the baseline of toxicity: GOT, GPT, BUN, creatinine, uric acid
   iii. Anemic condition: hemoglobin and hematocrit

(b) send 1 red top and 1 green top tubes to Taipei Institute of Pathology for testing toxicity of heavy metals: mercury, arsenic, lead, cadmium, and copper, and the test of iron and TIBC

4. **Study nurse 賴美齡**

(a) contacts subjects
(b) arranges clinic visits for subjects: registration, lead subjects to the clinic, etc.
(c) administers questionnaires (Appendix 8)
(d) gives out Motrin, study products, and copied menstrual record (Appendix 9), and diary (Appendix 10)
(e) takes information on emergency or any medical treatment for which women seek on their own
(f) makes copy of charts and stores at the study center
(g) performs centrifugation of whole blood and aliquots all leftover blood specimens, and brings back to store at study center
(h) checks the completion of procedures and records in the logs (Appendix 7)
(i) enters data into database

5. **Research assistants**

(1) Research assistant (TBA)

(a) bags the study products, Motrin, menstrual record, and diary
(b) hands out the bags to the study nurse
(c) collects and record returned study products, menstrual record, and diary
(d) manages the inventory of study products on each subject
(e) manages files
(f) coordinates for the needs between study nurse and subjects
(g) arranges meetings
(h) enters data and manages database

(2) Research assistant 辛毓真

(a) performs quality assurance of four-agents-decoction
(b) manages the stock room of study medicines
(c) helps bagging
(d) helps data entry and management

6. **Nursing teacher, 郝冬梅 at CTI and 謝伶俐 at TMC**
   (a) gives out Motrin on gynecologist's order to subjects need extra
   (b) traces students when necessary
   (c) collects information on emergency or other medical treatment that subjects have sought on their own

A. Grouped differentiated diagnosis will be performed one more time on Saturdays before follow-up period starts and sonography will be at the third treatment visit.

All the procedures used previously will be followed.

H. Follow-up—3 cycles (FV1, FV2, FV3)

1. **Gynecology examination**

Drs. 劉裕森 at CHH and 劉嘉燿 at TSGH
(a) performs check-up
(b) fills out the checklist of menstrual symptoms (Appendix 6)
(c) prescribes Motrin for 3 days for rescuing pain in next menstruation
(d) records all oral and written communications with other investigators and subjects
(e) supports care if emergency arises

2. **Study nurse 賴美齡:**
(a) contacts subjects
(b) arranges clinic visits for subjects: registration, lead subjects to the clinic, etc.
(c) administers questionnaires (Appendix 8)
(d) gives out Motrin, copied menstrual record (Appendix 9), and diary (Appendix 10)
(e) takes information on emergency or any medical treatment for which women seek on their own
(f) makes copy of charts and stores at the study center
(g) checks the completion of procedures and records in the logs (Appendix 7)
(h) enters data into database

3. **TCM examinations---Ancillary study**
   starting* from the 2nd follow-up visit*, clinics are set on Saturday mornings.

Drs. 陳建霖、張恒鴻、游智勝、蔡德豐、林高士
diagnoses of differentiated patterns

4. **Nursing teacher, 郝冬梅 at CTI and 謝伶俐 at TMC**

(a) gives out Motrin on gynecologist's order to subjects need extra
(b) traces students when necessary
(c) collects information on emergency or other medical treatment that subjects have sought on their own

(三)試驗期限與進度

An estimate of minimum 15 months is needed to complete this trial. The trial timetable cannot be fixed for the variations of menstrual cycle of participants. Briefly, the preparation will start in July 2002. The recruitment by using survey method will begin in September, 2002 when both returned students and freshmen start the school. Screening will be in the months of October and November, followed by wash-out and baseline periods in December and January. Treatment period will start right after Winter break for three cycles, which will be in the months of February through May, 2003. Follow-up will be from June through September, 2003.

(四)所需藥品或醫療器材名稱及數量

Study medications include Motrin (400mg) and study products: four-agents-decoction and placebo. After a preliminary discussions with three traditional Chinese Medicine practitioners individually in the study and an extensive review of literature (Appendix 13), the plan for the treatment has been set tentatively as having subjects take the study products starting seven days prior to and then until the onset of menstruation for one dose a day (26 capsules) and a booster after the end of menstruation for 5 days for each of the three cycles in the treatment period. Warm water should be used when swallowing the capsules. The final dosage will be decided by the investigators after reviewing the results from the survey.

Four-agents-decoction in capsules are made enough for 250 subjects from one batch of raw materials of the four agents: prepared Radix Rehmanniae praeparata, Radix angelicae Sinensis, Radix Paeoniae Alba, and Rhizoma Ligustici Chuanxiong. Each capsule contains 577 mg of powder. Twenty six capsules are needed to be filled for ~15 grams of the powders that are derived from 9 grams of concentrated decoction and 6 grams of starch as excipient from one dosage of raw materials. The number of capsules may be reduced to half, and the process of concentration and quality control of raw materials are under development with supervision offered by the Council of Traditional Chinese Medicine of DOH.

(五)病歷記錄用紙之格式

Please refer Appendices 6-10.

(六)資料之蒐集處理評估及統計分析方法

A. Sample Size Calculation

This is a pragmatically oriented pilot trial (20) incorporated with an epidemiologic study. Pain improvement of the two groups was estimated in this

pilot study using the data on analgesics for dysmenorrhea in Western medicine. Based on literature on clinical trials of analgesics, the average of pain improvement assessed by response rate was 70% in the treatment group and 30% in the placebo group. This estimate provides 90% power with 5% type I error, the calculated sample size is 56 subjects. With a 25% dropout rate, at least 100 subjects needed to be enrolled into this trial.

B. Randomization and Blindness

Subjects will be grouped into two strata by hospital, CHH for CTI students and TSGH for TMC students, respectively. Within each stratum, the subjects will then be randomized into treatment group or placebo group (Appendix 14). A permuted block randomization scheme is employed to generate random codes and an approximate equal number of subjects for the two treatment groups will be resulted. A list of treatment assignment linked with case number will be generated and kept by the PI and the study statistician. The codes and treatment assignment will not be released to any subjects, staff, and investigators other than those mentioned until the completion of data analysis.

C. Outcome Measures

From main study:

1. Diary
   (1) Pain severity and duration for the day
   (2) Reactions when taking capsules
   (3) Amount of analgesics used for rescuing the pain
   (4) Symptoms other than menstrual pain
   (5) Other medications
2. Menstrual record
   (1) Menstrual cycle including the dates and heaviness of menses from the first day of the trial
   (2) Absence from school
   3. Scores of individuals from the ancillary study
2. Questionnaires for pain improvement
3. Blood biochemical measures

From ancillary studies

Variables listed in the Appendix 16-17 will be studied for the relationships with the variables listed in the main studied.

No measure will be estimated by any investigator. All measures will be stratified by the treatment group and the comparisons will be made by groups as well as by changes within individuals by descriptive statistics, parametric or non-parametric analyses, depending on the data type and distribution.

D. Database

A database designed for easy use and reducing errors to minimum in the process of data entry has been proposed to the senior researchers in the division in hope of having it be constructed by the Information Technologist who was newly hired by this division. The database will be located in the study center—710, Nan

Kang campus of NHRI. If the idea of having this kind of database cannot be fulfilled, then Excel spreadsheet will be used instead as the means for data storage.

E. Data Management and Analysis

(1) Method of intent to treat is selected for data analysis.
(2) All data will be stratified by treatment group and examined separately. The data will also be evaluated for hospital effect.

The data on characteristics of subjects are extracted out from the first questionnaire. The data of all variables listed in the questionnaires, menstrual record, diary, and checklist of differentiated diagnosis are coded and entered into database by a RA (TBA). All data files will be collated for analysis. The datasets will be reviewed by all investigators, and the access of data will be accommodated to all investigators after sending in a 1-2 pages of proposal for documentation, which should contain the purpose of the access to data, the rationales and methods of the proposed study, and expected results from the study. Statistical analyses will be performed or supervised by the PI and the study statistician using SAS version 8.2 or other statistical softwares.

F. Efficacy and Safety Evaluation

Both diary and menstruation record are filled out before bedtime by subjects.

Primary efficacy endpoint: pain improvement

Baseline pain score: the score obtained at baseline, or the average score of previous cycles if no pain strikes before baseline cycle.

Pain duration: pain occurs from a day prior to until the second day after the onset of bleeding

Pain intensity:

1. the total VAS pain score from a day prior to until the second day after the onset of bleeding
2. the highest VAS pain score (peak of pain) from a day prior to until the second day after the onset of bleeding
3. the mean VAS scores from a day prior to until the second day after the onset of bleeding

The three outcomes will also be evaluated by extended measurement of the pain in the entire menstrual cycle.

If a subject discontinues taking the study products but remains to keep up clinic visits will be included in final analysis. To assess placebo effect, subjects are instructed to answer a question on guess of their study assignment in the questionnaire administered at the last follow-up visit and to list the reasons of the guess. The guess could be based on the appearance, taste, and smell of the study products, side effects, or effectiveness of the treatment (Appendix 8). The group of women who make correct guess will be analyzed separately.

The two treatment groups are compared by evaluating pain duration and pain intensity: visual analog scales (VAS), 4-point pain scale, and the amount of consumed analgesics at each of the three intervention and the three follow-up

periods. There are following ways for evaluation of pain improvement or positive response:

1. **Pain duration and intensity of the groups**
   Continuous data: mean and standard deviation, as well as 95% confidence intervals, of pain duration in hour and visual analog scales in mm.
   Categorical (ordinal) data: frequencies.
   (1) duration in hour is stratified into categorical outcome
   (2) VAS in mm is stratified into categorical outcome.
   (3) 4-point pain scale, which remains as in a scale from 1 to 4
2. **Changes in pain intensity within individuals**
   Continuous data: means and standard deviation of the difference in VAS in mm between baseline and assessed cycle
   Dichotomous data: response rate by the order of cycles
   A response of "yes" is defined by (1) positive difference in VAS in at least 18 mm or (2) by a decrease of one scale in categorized pain scales, and "no" by the difference in opposite direction.
   If a subject has menstrual pain, regardless of the intensity, in the consecutive five cycles from wash-out to treatment and no pain experienced in two of the three cycles of follow-up periods, then a positive response by her will be taken into account, the frequency of the positive responses by treatment group will in turn be assessed.
3. **Amount of consumed analgesics**
   A decrease in use of analgesics is to be considered as an efficacy of four-agents-decoction in the treatment of primary dysmenorrhea. Group comparisons as well as the changes within individuals by treatment groups for the amount of consumed analgesics will be performed.

In addition, odds ratios and the corresponding confidence intervals will be evaluated to compare the response rate between treatment groups. Repeated analysis of variance will be used to analyze the effects of treatment within and between groups over the trial period for the outcomes. Adjustment will be made where it is necessary. GEE method will be explored for further evaluation of the efficacy with the above first three approaches, proposed by the study statistician.

Secondary Efficacy endpoint: Association between response and differentiated diagnosis

There will be several patterns derived from the scoring system specified in the Ancillary study which may or may not be ordinal. For each treatment groups, the association between response and the assigned differentiated pattern will be explored by the Fisher's exact test. Appropriate statistical procedures will be determined after discussions among investigators over the final completed dataset.

Safety

The incidence of adverse events, abnormal laboratory values or treatment related toxicity will be summarized by treatment group. The proportions and the

95% confidence intervals will be given. The Pearson's chi-square test and Mantel-Haenszel method will be used to compare the incidence or ordered qualitative outcomes of the two regimens.

G. Interim Analysis and Post Hoc Analysis

Interim analysis is not considered in this trial.

If the result of this trial shows positive response, then a dose-response relationship will be explored using stratification by the length of treatment period. Risk factors for dysmenorrhea will be evaluated using the information in the questionnaires from the survey. The frequencies of the number of capsules taken during the treatment period, the types of differentiated diagnosis and food frequency from the survey are compared with the stratified VAS scores, as well as with 4-point pain scales for the assessment of the adequacy of dosage of pain relief and the association with differentiated diagnosis. The adjusted means of continuous data listed in VII-F will be obtained by adjusting for the amount of consumed analgesics. The Degree of agreement among the five Chinese medicine physicians for differentiated patterns will be evaluated by Kappa statistics. The diagnosis scores will be stratified and evaluated for the relationships with the listed variables in VII-F.

(七)問卷內容（若計畫涉及問卷調查）

Please refer Appendix 2. This questionnaire has been reviewed and approved by the IRB.

七、臨床上不良反應及處理方法：

Ingesting 26 capsules (may be less) per day does not lead to choking, aspiration, or even death, and no severe adverse event or reaction from the study product is anticipated (5). Women are asked to record any discomfort or sickness in diary (Appendix 10) to track the events between administration of the medicine and the adverse reactions.

In the case of emergency or illness, women are asked to seek medical treatment on their own and notify the PI. The women will provide the information to the study nurse or the nursing teacher for record. Consultation from the study physicians will also be available when requested. The study physicians will then decide whether the illness is related to study products.

Subjects are asked to practice absenteeism and avoid becoming pregnant. In the case of pregnancy, the women are asked to discontinue.

八、主持人及協同研究人員之學經歷、著作及所受訓練之背景資料:

Please refer Appendix 15.

九、受試者說明及同意書格式：

Please refer Appendix 4 and the one in NHRI format.

十、前人體試驗參考資料：

Please refer Appendices 13-1 and 13-2.

十一、出產國及核准上市國最高衛生主管機關許可製售證明影印本:

The certificate will be obtained from the manufacture once the company is identified.

十二、如尚屬研究中之新藥或新醫療器材，應說明其現況，並檢附生產國及其他國家核准進行人體試驗之證明文件影印本：

Not applicable.

十三、身心上可能產生之危險性與獲得之利益：

Potential Benefits

The direct benefits to the subjects are the opportunity of obtaining the first hand evidence on the efficacy of four-agents-decoction in capsules, differentiated patterns (or scores), and their relationships.

Potential Risks

The risk for the subjects is minimal, if there is any. If subjects experience breakthrough menstrual pain, then analgesics as rescue medicine are supplemented.

十四、財務上可能產生之危險與獲得之利益：

受試者赴醫院的十次門診、超音波檢查、血液檢驗、藥品、和兩次中醫體質辨証，都不需要受試者付費，同時我們會為受試者請准公假和付給受試者一百元作為公車和誤餐費。這次試驗後會有四物湯膠囊對經痛療效的初步證明，得到此項試驗結果的第一手資料和得知受試者的確切體質將會是受試者參加此一試驗研究的最大利益，而受試者在此試驗所需付出的將只是按時赴診和服用試驗品膠囊，填寫問卷，與給予兩次抽血。如受試者遇有任何因服用試驗品所產生的不預期反應，我們會依中華民國政府制定的臨床試驗醫療法為受試者處理。在試驗期間如受試者無法繼續，受試者可以隨時聯絡計畫總主持人，告知要終止參與。

十五、利益衝突：

Not applicable.

十六、其他資料：

Please refer the whole set of protocol for details.

國家衛生研究院人體試驗醫學倫理委員會
人體試驗受試者說明暨同意書

（**請參考計劃書中 Appendix 4 的口語化且具連貫性的說明書**）

（本書表將由計畫主持人親自向受試者說明詳細內容，並請受試者經過慎重考慮後方才簽名）

■藥品 □醫療器材 □醫療技術 □其他 ________倫委會編號：

本院現正進行一以隨機雙盲臨床試驗評估四物湯膠囊治療經痛的療效 __________研究計劃，邀請您參與此研究。本說明提供您有關本研究之相關資訊。研究主持人或其指定之代理研究人員（或其他協同主持醫師）將會爲您說明研究內容並回答您的任何疑問。

研究計畫名稱：

中文：以隨機雙盲臨床試驗評估四物湯膠囊治療經痛的療效

英文：Intervention Trial of Four-Agents-Decoction for Dysmenorrhea in Young College Women

主要主持人：葉梁蘭蘭　　　　聯絡電話：2652-4093

研究計畫贊助者：NHRI

受試者姓名：	年齡：
聯絡通訊地址：	
電話：	案歷號碼：
電子郵件：	

引言：

請您在同意參加本研究前，詳細閱讀這份同意書，我們願意隨時回答您任何相關問題。

四物湯自宋代以來就被廣泛地用來治療婦人的病痛，為了能了解老祖宗留傳下來的這帖古老方劑，我們這一群對四物湯有興趣的研究人員，將用已存在的臨床科學方法來驗證四物湯的療效。妳的參與成為受試者，

將會對此項研究的結果具有舉足輕重的重要性。

一、試驗目的：

此項研究是試驗四物湯對經痛的療效。

二、試驗方法與程序：（受試者標準、受試者數目、試驗進行方法、分析方法）

受試者是身受經痛苦楚的十八歲以上的大學女生。她們將被分成兩組，一組為四物湯組，另一組是安慰劑組。所用試驗品是膠囊劑型，由符合政府認證藥品優良製造規範 (GMP) 的中藥製造廠生產。在妳同意參加此一人體試驗後，我們將會以隨機分派方式，將妳分到其中一組，妳和進行試驗的醫師和護士都不知道妳所服用的膠囊內是否有四物湯。

這個試驗一共分成四階段:準備、篩選、治療與追蹤；所需的時間大約是十個月。在妳每次月事完畢後，到指定的醫院接受門診檢查，期間妳需要赴醫院十至十一次，接受研究人員對妳的月經週期做緊密觀察。

首先，你需要停止使用所有止痛藥物與方法，以為此試驗做準備；期間我們會提供止痛藥給妳，作為緩解妳的經痛之用。在經過一次完整的經期之後，我們會安排妳到醫院，接受婦科超音波和體外檢查，以便確定妳的經痛是為原發性。然後，是中醫的群辨証。這是由三位資深且權威的中醫師在星期六分別為妳的體質做一評估並作記錄。接下來的一次門診，我們會要在妳的手臂上抽取~35cc 血液，作為檢驗妳的肝、腎功能，貧血狀況，以及過去遭受重金屬污染的情況；同時我們會以這些檢驗值，再次確定妳參與此次臨床試驗的適合度。當門診完畢時，妳會領取到 3 天的止痛藥，用以作為緩解下一次經痛之用。

接著是治療期，也就是說，妳要在往後的三次經期前的七日開始直至月經來潮和經淨後的五天服用試驗品；服用的方式是早餐以前和夜晚睡覺以前以溫開水吞服。每一次經期所需服用的試驗品和作為緩解用的止痛藥，也同樣地是在每次看完中醫科和婦科門診時領取。在第三次治療門診時，我們會再做一次超音波檢查，並在妳的手臂上抽取~30cc 血液，進行與上次相同的檢驗，以便評估妳對試驗品的反應。

最後是追蹤期，妳會有三次婦科門診以及在每次結束時領取止痛藥。在第一次追蹤門診以前，三位中醫師又會分別為妳的體質再做評估並作記錄，以為確定妳的體質。

三、身心上可能導致之副作用、不適或危險：

疼痛難忍或是有腹瀉、火氣大、嘴巴乾、嘴角生瘡、經血過多或過長而感虛弱等

四、預期試驗效果：

這次試驗後會有四物湯膠囊對經痛療效的初步證明。

五、其他可能之治療方法選擇及說明：

在整個試驗期間，妳不能有性行爲。妳除了服用試驗品之外，不要服用口服避孕藥，也不要自行使用其他解除經痛的藥物或方法。如果經痛難忍，妳可以服用我們簽發給妳的止痛藥，以作爲緩解。

六、其他可能之損失或利益：

這次試驗後會有四物湯膠囊對經痛療效的初步證明，得到此項試驗結果的第一手資料和得知妳的確切體質將會是妳參加此一試驗研究的最大利益，而妳在此試驗所需付出的將只是按時赴診和服用試驗品膠囊，填寫問卷，與給予兩次抽血。

七、參加本研究計畫受試者個人權益將受到保護：

1. 如妳遇有任何因服用試驗品所產生的不預期反應，我們會依中華民國政府制定的臨床試驗醫療法爲妳處理。妳赴醫院的十次門診、超音波檢查、血液檢驗、藥品、和兩次中醫體質辨証，都不需要妳付費，同時我們會爲妳請准公假和付給妳一百元作爲公車和誤餐費。

妳所提供的所有記錄和臨床資料，將被視做機密，存放在上鎖的檔案櫃和房間裏；亦即妳的姓名不會出現在數據庫或任何的分析資料與報表中，除了計畫總主持人以外，無人可以指認出與妳有關的資料。如妳想持有自己的資料，我們會依妳的書面要求在此項臨床試驗研究結束後免費寄送給妳。如果有後續的研究需要使用到妳的資料和血液時，我們會在徵求到妳的同意之後使用。

2.

若疼痛難忍或是有腹瀉、火氣大、嘴巴乾、嘴角生瘡、經血過多或過長而感虛弱等，以致必須尋求醫療解除，妳可自行前往急診室求診或告知計畫總主持人-葉梁蘭蘭博士，電話為 2652-4093，為妳安排診療。

八、可自由選擇是否參與研究：

Not Applicable.

(Because of the nature of this trial, subjects will be arranged to have the opportunity to discuss all trial-related issues with physicians (investigators) at workshop before sign the Informed Consent and make commitment.)

如對您之權利有任何疑問，請電詢本院人體試驗醫學倫理委員會，電話爲：？？？

九、撤回同意，退出試驗：

在試驗期間如妳無法繼續，妳可以隨時聯絡計畫總主持人-葉梁蘭蘭博士，電話為 2652-4093

您亦可聯絡本院人體試驗醫學倫理委員會，電話爲：？？？？

十、機密性：

妳所提供的所有記錄和臨床資料，將被視做機密，存放在上鎖的檔案櫃和房間裏；亦即妳的姓名不會出現在數據庫或任何的分析資料與報表中，除了計畫總主持人以外，無人可以指認出與妳有關的資料。如妳想持有自己的資料，我們會依妳的書面要求在此項臨床試驗研究結束後免費寄送給妳。如果有後續的研究需要使用到妳的資料和血液時，我們會在徵求到妳的同意之後使用。

？？　本院將在法律所規範之程度內視受試者之資料爲機密，您的姓名將被一個研究的編號取代，您的隱私將會謹慎的保護。您亦瞭解本院人體試驗醫學倫理委員會（或研究贊助者、衛生署）皆有權檢視受試者的資料。

？（例）1.本試驗結果數據除發表於科學性刊物外，不會對外公開。所有刊登出來的文章，也不容許出現任何可資辨認受試者之資訊。

? 2.本試驗之結果，將只發予受試者本人；惟基因報告部分，需時較久，如有具體結果時，會主動通知您。

您的選擇：（□要，□不要）基因報告

? 3.您可選擇是否容許以後其他研究利用本計畫所得基因資料（屆時所有可供辨識個人身分之資訊將被刪除）。

我的基因資料（□可以，□不可以）被其他研究使用

? 4.本計畫所得之基因資料，如果受試者日後不同意繼續提供分析使用，可以隨時以書面通知計畫主持人停止使用。

? 5.您是否同意您的血液、尿液樣本用於其他醫學研究？

您的選擇：□同意，□不同意

? 6.本研究結束時，若您的 DNA 樣本有剩餘時，您可以選擇您的 DNA 標本處理方法。

您的選擇：□歸還，□銷毀，
□提供其他相關基因研究
（屆時會再請您簽署另一份同意書。）

十一、利益衝突：

? ? ? ? ? ?

十二、簽章

(一)□主持人
□協同主持人
□研究代理人 ________________________（請簽名）

已詳細解釋有關本研究計畫中上述研究方法之性質與目的，及可能產生之危險與利益。

本院總計畫主持人________________（簽章）日期：-----------------

主治醫師________________（簽章）日期：---------------

(二)本人已詳細瞭解上述研究方法及其所可能產生之危險與利益，有關本試驗計畫之疑問，業經計畫主持人詳細予以解釋。本人同意接受爲人體試驗計畫之自願受試者。

受試者或代理人簽章：____________________日期：______________

如您是受試者代理人，請用正楷書寫您的姓名：並指出您是受試者之：
□法定代理人 □監護人 □配偶 □成年子女 □父母 □兄弟姊妹
□受任人（需附委任書）□其它，請說明

(三) 見證人簽名：________________ 日期：______________

The following is the format I wrote:

我已經了解此試驗的目的，進行方法和可能獲得的利益與承擔的風險，經過仔細考慮之後，我同意參加四物湯膠囊療效的人體試驗。

受試者簽名____________________日期___________________

如未滿 20 歲，□父 □母 □監護人 □法定代理人

姓名____________________

簽名____________________日期___________________

計畫主持人簽名________________日期___________________

姓名____________ 案例號 ____________

Appendix 4

人體試驗四物湯膠囊療效之受試者說明暨同意書

妳被邀請參加評估四物湯對經痛療效的臨床試驗，如有任何疑問，請致電 郝冬梅老師 2782-1862 或者是計畫主持人 葉梁蘭蘭博士 2652-4093。

每月的經期來潮是一種正常的生理現象，然而最近調查顯示約有 84％的女性會遭受經痛的苦楚。經痛的程度可從隱約的小腹痛，到腹部的刺痛，更甚者會有嘔吐或腹瀉發生以致必須臥床休息。

四物湯自宋代以來就被廣泛地用來治療婦人的病痛，然而其療效尚未以科學方法評估過。為了能了解老祖宗留傳下來的古老方劑，我們這一群對四物湯療效有興趣的研究人員，將用已存在的科學方法來驗證四物湯的療效。妳的參與成為受試者，將會對此項研究的結果具有舉足輕重的重要性。

此項研究是測試四物湯對經痛的療效，受試者將被分成兩組，一組為四物湯組，另一組是安慰劑組，所用試驗品是裝填在由符合政府規格的 GMP 製造廠出產的濃縮膠囊中。在妳同意參加此一人體試驗後，我們將會以抽籤方式，將妳分到其中一組，妳和進行試驗的護士及研究助理都不知道妳所服用的膠囊內是否有四物湯。

這個試驗所需的時間一共是三個月。在試驗開始時，三位中醫師將會為妳的體質做一評估並作記錄，然而評估的結果，將要等到試驗結束後，與膠囊的內容一併寫成報告遞交給妳。

在三個月的試驗期裡，妳每天在研究人員面前服用 26 粒膠囊，這是約等於一帖四物湯煎煮出的濃縮湯量。因此妳是以較簡便的方法服用等劑量的四物湯。

這計畫的程序是每一天妳將會收到一盒試驗品，內含當日的膠囊量，以及一張臨床記錄表和一張常用食物調查表。當日的中餐前和放學前

以溫開水吞服，並將這一天的臨床資料與研究人員一起填妥。常用食物調查表則隨身攜帶填答，次日交回。如有未服完的膠囊，帶回家在睡覺前服用；如果仍然有未服完的膠囊，則留置盒中，次日交給研究人員收回該盒，同時領取另一天的試驗品與兩張記錄表。遇假日時，研究人員會將所需服用的試驗品量和所需填寫的記錄表一併於假日前一天交給妳帶回，用過的盒子，未用完的膠囊以及記錄表則在放假之後的第一天交給研究人員。

服用期間，如仍感經痛，我們為妳準備了止痛藥，妳可依疼痛的程度或需要的程度服用。除了服用試驗品和止痛藥之外，在這段試驗期間，妳不要服用口服避孕藥，也不要自行使用其他解除經痛的藥物或方法。若須要醫療或是有腹瀉、火氣大，嘴巴乾，嘴角生瘡等，請告知研究人員為妳安排診療，事後務必在臨床記錄表內記錄妳所服用的藥名和藥量。

在這段試驗期間，如妳無法繼續，妳可以隨時聯絡計畫主持人-葉梁蘭蘭博士，電話為 2652-4093，告知妳要終止妳的參與。在三個月試驗終止時妳將會由同樣的三位中醫師再做一次體質評估。往後的三個月，研究人員並將會再以信件與妳聯絡，追蹤妳的經痛情形。

在這次試驗期間，研究人員在每一個月的最後一個星期六會與所有受試者聚會一次，大家可以交換意見與心得，並且在當天妳會收到 250 元的零用金。

這次試驗將可提供四物湯膠囊療效的初步證明，得到這結果的第一手資料將會是妳參加此一試驗研究的最大利益，而妳在此試驗所需付出的將只是每天服用膠囊與填寫兩張記錄表的時間。如妳遇有任何因服用試驗品所產生的不預期反應，藥廠已購有保險來負責妳的醫療費用。

妳所提供的所有臨床記錄，將視做機密，存放在上鎖的檔案櫃裏；亦即妳的姓名不會出現在數據庫或任何的分析資料與報表中，除了計畫主持人以外，無人可以指認出與妳有關的資料。如妳需要妳自己的資料，我們會依妳的書面要求在此項研究結束後免費寄送給妳。

我已經了解此試驗的目的與進行方法，經過考慮之後，我同意參加四物湯膠囊療效的人體試驗。

受試者簽名____________________日期___________________

如未滿 20 歲，☐父 ☐母 ☐監護人 ☐法定代理人

簽名______________________日期____________________

計畫主持人簽名___________________日期__________________

變更人體試驗計畫申請書明細

計畫名稱：以隨機雙盲臨床試驗評估四物湯膠囊治療年輕女大學生經痛的療效

變更頁次：計畫書中第十二頁

變更計畫原因：本計畫原本規劃三次治療週期，然因治療期間正值冬季，而依中醫理論，痛經的起因是「不通則痛」，其基本標證為氣血不通；又第三次治療週期適逢春節假日與寒假假期，部分受試者的飲食作息可能會有所改變，以致不易準確的評估出體質與試驗藥物的療效，在經過與中、西醫師們討論後，決議需追加一次治療週期。

國家衛生研究院人體試驗醫學倫理委員會
人體試驗受試者說明暨同意書

■藥品 □醫療器材 □醫療技術 □其他

妳已參與本院進行一以隨機雙盲臨床試驗評估四物湯膠囊治療年輕女大學生經痛的療效之研究計劃。本說明提供妳有關本研究【變更治療期】之相關資訊。研究主持人或其指定之代理研究人員（或其他協同主持醫師）將會為妳說明研究內容並回答妳的任何疑問。

原研究計畫名稱：

中文：以隨機雙盲臨床試驗評估四物湯膠囊治療年輕女大學生經痛的療效

英文：**Randomized Double-blind Clinical Trial of Four-Agents-Decoction in Capsule for Dysmenorrhea in Young College Women**

總主持人：梁蘭蘭博士 臨床研究組助研究員　　聯絡電話：2652-4093

試驗研究計畫贊助者：國家衛生研究院

試驗執行單位：國家衛生研究院及三軍總醫院與台北市立忠孝醫院

合作與施行檢查醫院： 1. 三軍總醫院— 臺北市內湖區成功路二段 325 號

2. 台北市立忠孝醫院—臺北市南港區同德路 87 號

協同主持人：

國家衛生研究院：蘇益仁醫師，臨床研究組組主任

丘政民博士，生物統計與生物資訊研究組助研究員

三軍總醫院：劉嘉燿醫師，婦產部主治醫師

林高士醫師，中醫部主任

王麗香醫師，中醫部主治醫師

台北市立忠孝醫院：劉裕森醫師，婦產科主治醫師

蔡德豐醫師，中醫科主任
私立長庚中醫分院：邵秉家醫師，婦兒科主治醫師

如今妳已經完成了「以隨機雙盲臨床試驗評估四物湯膠囊治療年輕女大學生經痛的療效」臨床試驗原本計畫中之三次治療週期，然因治療期間正值冬季，而依中醫理論，痛經的起因是「不通則痛」，其基本標症爲氣血不通；又第三次治療週期適逢春節假日與寒假假期，妳的飲食作息可能會有所改變，以致不易準確地評估出妳的體質與試驗藥物—四物湯－的療效，因此將要追加一次治療週期，換句話說，這次臨床試驗療程將由三次改變爲四次治療週期。

在服用第四次療程的試驗藥物膠囊時，妳將會依原計畫之時程，被再次安排進行體質辨證分型和超音波診察，以及在妳的手臂上抽取~20ml 血液做生化值的複檢。之後，妳將會被追蹤三次月經週期和經痛的程度，期間，妳將只能使用 Motrin（400mg）做爲唯一解緩經痛的方法，並且繼續秤量棉墊和記錄來經情形。

簽章

(一)　協同主持人---中醫師

________________________（簽名）日期：________

服務單位：

協同主持人---婦產科醫師

________________________（簽名）日期：

服務單位：

已詳細解釋有關改變本研究計畫中上述研究方法之性質與目的，及可能產生之危險與利益。

(二)　本人已詳細瞭解上述研究方法及其所可能產生之危險與利益，

有關本試驗計畫之疑問，業經計畫主持人詳細予以解釋。
本人同意接受以上的追加治療。

受試者簽名：____________________日期：_______________

代理人簽名：____________________日期：_______________

如妳是受試者代理人，請用正楷書寫妳的姓名：並指出妳是受試者之：

□法定代理人 □監護人 □父母 □兄姊 □受任人（需附委任書）□其它，請說明

計畫主持人______________________（簽名）日期：
