# Supplementary material for: A Randomised Placebo-Controlled Trial of a Traditional Chinese Herbal Formula in the Treatment of Primary Dysmenorrhoea
Source: PLoS One. 2007 Aug 15;2(8):e719. doi: 10.1371/journal.pone.0000719 (PMC1940310; doi:10.1371/journal.pone.0000719)
Supplement: Checklist S1 — CONSORT Checklist (0.11 MB DOC) [file pone.0000719.s004.doc]

**CONSORT Checklist of items to include when reporting a randomized trial**

| PAPER SECTION And topic | Item | Description | Reported on  Page # |
| --- | --- | --- | --- |
| *TITLE & ABSTRACT* | 1 | [How participants were allocated to interventions](http://www.consort-statement.org/examples1.htm) (*e.g*., "random allocation", "randomized", or "randomly assigned"). | 1-4 |
| *INTRODUCTION* Background | 2 | [Scientific background and explanation of rationale.](http://www.consort-statement.org/examples2.htm) | 5-7 |
| *METHODS* Participants | 3 | [Eligibility criteria for participants](http://www.consort-statement.org/examples3a.htm) and the [settings and locations where the data were collected](http://www.consort-statement.org/examples3b.htm). | 8-9 |
| Interventions | 4 | [Precise details of the interventions intended for each group and how and when they were actually administered.](http://www.consort-statement.org/examples4.htm) | 9-11 |
| Objectives | 5 | [Specific objectives and hypotheses](http://www.consort-statement.org/examples5.htm). | 12 |
| Outcomes | 6 | [Clearly defined primary and secondary outcome measures](http://www.consort-statement.org/examples6a.htm) and, when applicable, any [methods used to enhance the quality of measurements](http://www.consort-statement.org/examples6b.htm) (*e.g.*, multiple observations, training of assessors). | 12-14 |
| Sample size | 7 | [How sample size was determined](http://www.consort-statement.org/examples7a.htm) and, when applicable, [explanation of any interim analyses and stopping rules](http://www.consort-statement.org/examples7b.htm). | 14 |
| Randomization -- Sequence generation | 8 | [Method used to generate the random allocation sequence, including details of any restrictions](http://www.consort-statement.org/examples8a.htm) (*e.g*., blocking, stratification) | 14-15 |
| Randomization -- Allocation concealment | 9 | [Method used to implement the random allocation sequence](http://www.consort-statement.org/examples9.htm) (*e.g*., numbered containers or central telephone), clarifying whether the sequence was concealed until interventions were assigned. | 15 |
| Randomization -- Implementation | 10 | [Who generated the allocation sequence, who enrolled participants, and who assigned participants to their groups.](http://www.consort-statement.org/examples10.htm) | 15 |
| Blinding (masking) | 11 | [Whether or not participants, those administering the interventions, and those assessing the outcomes were blinded to group assignment.](http://www.consort-statement.org/examples11a.htm) When relevant, [how the success of blinding was evaluated](http://www.consort-statement.org/examples11b.htm). | 15-16 |
| Statistical methods | 12 | [Statistical methods used to compare groups for primary outcome(s)](http://www.consort-statement.org/examples12a.htm); [Methods for additional analyses,](http://www.consort-statement.org/examples12b.htm) such as subgroup analyses and adjusted analyses. | 16-17 |
| *RESULTS*  Participant flow | 13 | [Flow of participants through each stage](http://www.consort-statement.org/examples13a.htm) (a diagram is strongly recommended). Specifically, for each group report the numbers of participants randomly assigned, receiving intended treatment, completing the study protocol, and analyzed for the primary outcome. [Describe protocol deviations from study as planned, together with reasons.](http://www.consort-statement.org/examples13b.htm) | 18 |
| Recruitment | 14 | [Dates defining the periods of recruitment and follow-up.](http://www.consort-statement.org/examples14.htm) | 18 |
| Baseline data | 15 | [Baseline demographic and clinical characteristics of each group.](http://www.consort-statement.org/examples15.htm) | 18-19 |
| Numbers analyzed | 16 | [Number of participants (denominator) in each group included in each analysis and whether the analysis was by "intention-to-treat"](http://www.consort-statement.org/examples16.htm). State the results in absolute numbers when feasible (*e.g*., 10/20, not 50%). | 19 |
| Outcomes and estimation | 17 | [For each primary and secondary outcome, a summary of results for each group, and the estimated effect size and its precision](http://www.consort-statement.org/examples17.htm) (*e.g.*, 95% confidence interval). | 19-22 |
| Ancillary analyses | 18 | [Address multiplicity by reporting any other analyses performed](http://www.consort-statement.org/examples18.htm), including subgroup analyses and adjusted analyses, indicating those pre-specified and those exploratory. | 22-23 |
| Adverse events | 19 | [All important adverse events or side effects in each intervention group.](http://www.consort-statement.org/examples19.htm) | 23-24 |
| *DISCUSSION* Interpretation | 20 | [Interpretation of the results](http://www.consort-statement.org/examples20.htm), taking into account study hypotheses, sources of potential bias or imprecision and the dangers associated with multiplicity of analyses and outcomes. | 25-28 |
| Generalizability | 21 | [Generalizability (external validity) of the trial findings.](http://www.consort-statement.org/examples21.htm) | 28 |
| Overall evidence | 22 | [General interpretation of the results in the context of current evidence.](http://www.consort-statement.org/examples22.htm) | 28-30 |
